# SARS-CoV-2 is detectable using sensitive RNA saliva testing days before viral load reaches detection range of low-sensitivity nasal swab tests

**DOI:** 10.1101/2021.04.02.21254771

**Published:** 2021-04-07

**Authors:** Emily S. Savela, Alexander Winnett, Anna E. Romano, Michael K. Porter, Natasha Shelby, Reid Akana, Jenny Ji, Matthew M. Cooper, Noah W. Schlenker, Jessica A. Reyes, Alyssa M. Carter, Jacob T. Barlow, Colten Tognazzini, Matthew Feaster, Ying-Ying Goh, Rustem F. Ismagilov

**Affiliations:** 1. California Institute of Technology, 1200 E. California Blvd., Pasadena, CA, USA 91125; 2. City of Pasadena Public Health Department, 1845 N. Fair Oaks Ave., Pasadena, CA, USA 91103

## Abstract

Early detection of SARS-CoV-2 infection is critical to reduce asymptomatic and pre-symptomatic spread of COVID-19, curb the spread of viral variants by travelers, and maximize efficacy of therapeutic treatments. We designed a study to evaluate the preferred test sensitivity and sample type (saliva and nasal swab) for detecting early infections of COVID-19. We performed a case-ascertained study to monitor household contacts of individuals recently diagnosed with a SARS-CoV-2 infection. From those individuals, we obtained twice-daily self-collected anterior-nares nasal swabs and saliva samples and quantified SARS-CoV-2 RNA viral loads in those samples using high-sensitivity RT-qPCR and RT-ddPCR assays. We found that SARS-CoV-2 RNA first appears in saliva and then in nasal-swab samples. A high-sensitivity (limit of detection of ~10^3^ copies/mL) RNA test detected SARS-CoV-2 virus in saliva 1.5 to 4.5 days before the viral load in the paired nasal-swab samples exceeded the limit of detection of low-sensitivity tests. It was possible to observe a high (>10^7^-10^8^ copies/mL) viral load in saliva samples while the paired nasal swab was either negative or had low (~10^3^ copies/mL) viral load. Our results indicate that both sampling site and test sensitivity must be considered to ensure early detection of SARS-CoV-2 infection: high-sensitivity tests that use saliva can detect SARS-CoV-2 infection days earlier than low-sensitivity tests that use nasal swabs. Furthermore, early in the infection, low-sensitivity tests that use nasal swabs may miss SARS-CoV-2-positive individuals with very high and potentially infectious viral loads in saliva.

## Introduction

Early detection of SARS-CoV-2 infection is needed to reduce pre-symptomatic and asymptomatic transmission, including the introduction and spread of new viral variants from travelers. More than half of transmission events^1^ occur from pre-symptomatic or asymptomatic persons. Detecting infection early enables individuals to self-isolate sooner, reducing transmission within households and local communities. Diagnostics that can reliably detect the earliest stage of SARS-CoV-2 infection are also critical for protecting vulnerable populations, including individuals hospitalized for non-COVID-19 illnesses and individuals at higher risk for severe disease due to multiple medical comorbidities (e.g. residents of skilled nursing or long-term care facilities and mental-health institutes). Although vaccination is reducing severe COVID-19 outcomes, a sizable portion of the world’s population is likely to remain unvaccinated due to limited vaccine availability, medical ineligibility, or personal preference. For example, in the U.S., children under age 16, even those with significant health conditions or co-morbidities, are not yet eligible for vaccination. Thus, testing remains an important tool for preventing outbreaks among children in schools and daycares (where children under age 2 cannot wear masks), which may affect other high-risk and unvaccinated individuals. Tests that detect early infections are also important to prevent viral transmission among people living or working in congregate or crowded settings, including people living in college dormitories, people experiencing homelessness or incarceration, and children in summer camps.

Beyond outbreak prevention and control, early detection of COVID-19 is critical for individual patient care, as several treatments show exclusive or increased efficacy only when given early in the infection. The advantage of earlier treatment initiation is likely due to reduction of viral replication either directly or by promotion of an early effective inflammatory response, which prevents a later exaggerated inflammatory response.^2^ Results of the ACTT-1 trial demonstrated a survival benefit in patients for whom Remdesivir was initiated when receiving supplemental oxygen, but that benefit was lost once disease progressed and advanced respiratory support was needed.^2–4^ Convalescent plasma failed to show efficacy in a study where the median time to entry in the study was 8 days after symptom onset,^5^ but demonstrated protection against progression to respiratory failure when given to individuals of advanced age earlier in the course of the illness.^6^ Similarly, the use of anti-SARS-CoV-2 monoclonal antibody therapy (bamlanivimab or casirivimab plus imdevimab) did not show benefit over placebo in a cohort of hospitalized patients.^7^ However, when given to outpatients with mild or moderate COVID-19, who may have otherwise progressed to hospitalization later in the course of illness, reductions in emergency room or medical visit rate and more rapid declines in viral load^8–10^ have been observed. Further, a greater effect was observed among the subgroup of patients who had not yet developed a detectable endogenous antibody response.^2,9^

However, currently it is unclear how to detect SARS-CoV-2 infection at the earliest stages. Does one need a high-sensitivity test, or would a low-sensitivity test suffice? Which sample type should one use?

Tests with high analytical sensitivity can detect low levels of virus and molecular components of the virus, such as viral RNA or proteins, present in a sample. Analytical sensitivity is described by the limit-of-detection (LOD) of a test (defined as the lowest concentration of the viral molecules that produces 95% or better probability of detection). The lower the LOD, the higher the analytical sensitivity of the assay is. LOD values of SARS-CoV-2 diagnostic tests are described in various units; the most directly comparable among tests are units that report the number of viruses (viral particles) or viral RNA copies per milliliter of sample. Viral RNA copies/mL are roughly equivalent to genome equivalents/mL (GE/mL) or nucleic acid detectable units/mL (NDU/mL). These LOD values are tabulated by the U.S. Food & Drug Administration (FDA).^11^ High-sensitivity tests have LOD values equivalent to ~10^2^ to 10^3^ copies/mL of sample, whereas low-sensitivity tests have LOD values equivalent to ~10^5^ to 10^7^ copies/mL. Therefore, to choose the appropriate test for reliable detection, one needs to measure viral loads present in samples collected early in the course of infection, and then choose a test with an LOD below that viral load.

Initial data by us^12^ and others^13–15^ show that, at least in some humans, SARS-CoV-2 viral load can be low (in the range of 10^3^ -10^5^ copies per mL of saliva sample) early in infection and therefore high-sensitivity tests would be required to reliably detect infection. However, most previous studies to date have focused only on viral detection, not viral-load quantification. A few studies collect samples in RNA-stabilizing buffers,^16–19^ but most collect dry-swabs or saliva in sterile collection vessels.^15,20–31^ Without an RNA-stabilizing buffer, there is some risk of viral degradation during transport and handling. Moreover, most studies do not report quantitative analysis of viral loads, making it more difficult to interpret the observed results relative to the LOD values of the particular test used.

Sampling site or specimen type may also be critical to early detection. Other respiratory viruses have been shown to have detection rates that vary by sampling site,^32^ which have occasionally been linked to viral tropism: for example, the cellular receptor for entry of MERS-CoV is expressed nearly exclusively in the lower respiratory tract, prompting recommendations for diagnostic testing of specific sample types (bronchoalveolar lavage, sputum and tracheal aspirates).^33^ Limited data directly compare SARS-CoV-2 viral load in paired sample types early in the course of infection, as needed to inform optimal sampling site for early detection. Although nasopharyngeal swab is often considered the gold standard for SARS-CoV-2 detection, it requires collection by a healthcare worker and is not well tolerated. Other sample types, such as nasal (anterior-nares or mid-turbinate) swabs^21,24,26,34^ and saliva^20,35,36^ are more practical, especially for repeated sampling in serial surveillance testing (also described as “screening”).

The majority of studies that have compared multiple sample types for SARS-CoV-2 detection selected individuals known to be positive for SARS-CoV-2, thereby missing the early stages of infection. An excellent study comparing nasal swabs and saliva sampling early in the infection among adults at a university^15^ suggested that high-sensitivity testing is needed for early detection; however, these results were based on detection alone, and quantitative measurements of viral load early in the infection or raw data were not reported.

To understand the required test sensitivity and the optimal sample type for earliest SARS-CoV-2 detection, we designed a case-ascertained study of household transmission with high-frequency sampling of both saliva and anterior-nares nasal swabs. Building on our earlier work,^12^ we enrolled individuals from Los Angeles County, California, ages 6 and older who had recently tested positive (household index case) and exposed household contacts at risk of infection. All participants self-collected saliva and nasal-swabs twice daily, in the morning upon waking and before bed. Importantly, all samples were immediately placed in a guanidinium-based inactivating solution (see Methods) that preserves viral RNA until analysis. Samples were screened for positivity and monitored for sufficient amounts of human ribonuclease P (RNase P)^37^ indicating high-quality and consistent sample collection. If a transmission event was observed (a previously SARS-CoV-2 negative participant tested positive), viral load in samples prospectively collected from that participant were quantified via two independent methods through the early and full course of acute SARS-CoV-2 infection.

## Results

First, we established and validated two independent quantitative assays to measure SARS-CoV-2 viral load: a quantitative reverse-transcription PCR (RT-qPCR) based on the assay put forth by the U.S. Centers for Disease Control and Prevention (CDC),^37^ and a reverse-transcription droplet digital PCR (RT-ddPCR) assay developed by Bio-Rad. Both of these assays received an emergency use authorization (EUA) for qualitative, but not quantitative, detection of SARS-CoV-2. In initial testing, when combined with standard KingFisher MagMax sample preparation protocols, these assays performed well to quantify heat-inactivated SARS-CoV-2 viral particles spiked into commercially available SARS-CoV-2 negative saliva and nasal fluid from pooled donors. However, they did not provide reliable quantification when we analyzed individual saliva samples freshly collected from positive participants in this study. Carryover of materials from some of the mucus-rich samples was inhibitory, as determined by RT-ddPCR analysis of dilutions of eluted RNA (data not shown). We optimized the extraction and each quantitative assay protocol (see Methods) to obtain more reliable quantification of SARS-CoV-2 viral load.

**Figure 1.**
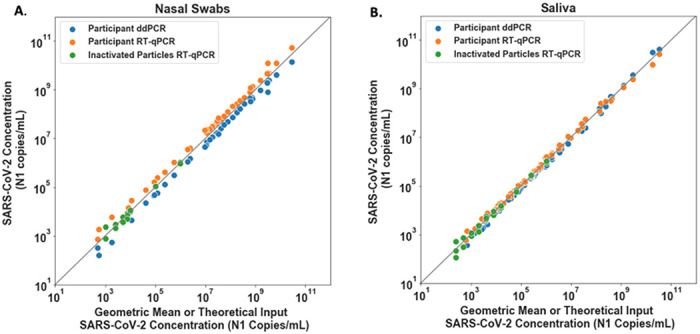
SARS-CoV-2 viral load quantification for nasal swab (A) and saliva (B) specimens from positive participants measured with RT-ddPCR and RT-qPCR. Participant nasal swab (A) or saliva (B) SARS-CoV-2 N1 concentration (copies/mL) per detection method, RT-ddPCR (Blue circles) and RT-qPCR (orange circles) plotted against geometric mean of RT-qPCR and RT-ddPCR viral load concentrations. A total of 42 nasal swab and 63 saliva samples from study participants were quantified with both methods. Theoretical SARS-CoV-2 concentration input represents data from calibration curves created with a dilution series of contrived samples prepared using commercial, inactivated SARS-CoV-2 particles spiked into commercially available SARS-CoV-2 negative saliva or nasal fluid pooled from human donors (green circles), extracted and detected with RT-qPCR. Grey line represents x=y.

We cross-validated our quantification methods in two steps. First, we used commercial, heat-inactivated SARS-CoV-2 viral particles to establish calibration curves for both saliva and swab samples to convert RT-qPCR quantification cycle values (Cq, also described as Ct) to viral load. Input particle concentrations for each point on the curve were calculated based on the stock quantification reported on the certificate of analysis for each lot of particles. We could not extend the calibration curve to very high viral loads because of the limited concentration of viral particle stock; so, to confirm performance at high viral loads, we quantified 42 swab and 63 saliva samples from SARS-CoV-2-positive participants with both RT-qPCR and RT-ddPCR methods. For each sample, we plotted on the logarithmic scale the results from each of the two independent quantitative assays against the geometric mean of these two results (Fig. 1). On the same plots, we also show the data from the experiments used to establish the calibration curves (Fig. S1). We observed excellent concordance between the calibration curve, RT-qPCR and RT-ddPCR assays over the entire dynamic range of input concentrations, even though RT-qPCR eluents were run as-is and RT-ddPCR eluents from high-concentration samples were significantly diluted. For nasal swab samples, RT-ddPCR values were slightly below the RT-qPCR values, however this difference was consistent across the entire dynamic range, indicating no concentration-dependent biases like enzymatic inhibition. We chose not to adjust the calibration curve to fit RT-ddPCR values; we reported the concentrations based on the calibration curves derived from the certificate of analysis from the BEI reference material. For saliva samples, all points tightly clustered around the x=y line. We confirmed that the LOD of the modified assay was 1,000 copies/mL or better (see Methods, Fig. S2).

**Figure 2.**
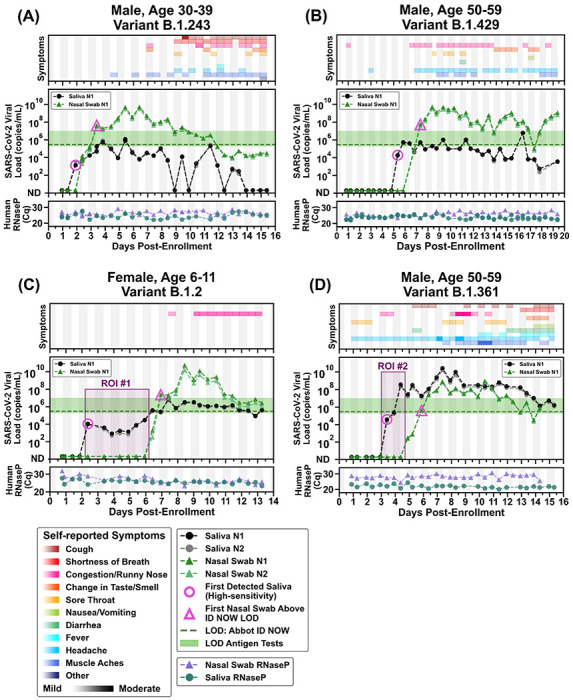
Symptoms and SARS-CoV-2 viral loads in paired saliva and nasal-swab samples of four participants who became SARS-CoV-2 positive during study participation. **(A-D)** Self-reported twice-daily symptom data over the course of enrollment are shown as a top panel for each of the participants (see color-coded legend for symptom categories). Viral loads are reported for the N1 and N2 genes of SARS-CoV-2 for both saliva (black and grey circles) and nasal swab samples (dark-green and light-green triangles); indeterminate results are not shown, ND = not detected for Cqs ≥40 (see Methods for details). The limit of detection (LOD) of the Abbott ID NOW (300,000 NDU/mL^11^) is indicated by the horizontal green dashed line; the range of LODs of antigen tests (horizontal green bar) are shown for reference (data are from Table S2 in ref. 12). A diagnostic test does not provide reliable detection for samples with viral loads below its LOD. For each participant, the first detected saliva point is emphasized with a pink circle and their first nasal swab point above the LOD of the ID NOW is emphasized with a pink triangle. Vertical shading in grey indicates nighttime (8pm – 8am). Internal control of RNase P Cqs from the CDC primer set are provided for each sample to compare self-sampling consistency and sample integrity (failed samples, where RNase P Cq ≥40, are not plotted). Participant gender, age range, and SARS-CoV-2 variant is given in each panel’s title. Two regions of interest (ROI) are indicated by purple-shaded rectangles and discussed in the main text.

Next, to quantify viral load at the earliest stage of infection, we analyzed the viral loads in the saliva and nasal swabs of participants who were negative in both saliva and nasal swab upon enrollment and became positive during their participation in the study (Fig. 2). We extended each participant’s enrollment in our study to acquire 14 days of paired saliva and nasal swab samples. All participants reported the presence and relative severity of any symptoms twice per day. Symptom trackers listed the most commonly reported COVID-19 symptoms^38^ (cough, shortness of breath, congestion, runny nose, change in taste/smell, sore throat, nausea, vomiting, diarrhea, fever, headache, and muscle/body aches). Participants could also write in any symptoms not listed. Symptom data were analyzed and plotted together with the viral load data vs time post-enrollment. For convenience, we marked the LOD of Abbott ID NOW (300,000 nucleic-acid-amplification-test-detectable units (NDU)/mL per FDA testing^11^) and the LOD range of antigen tests (one would not expect a test to reliably detect samples with viral loads below the test’s LOD). Because nasal swabs are commonly used with low-sensitivity tests, and because such tests are proposed to be utilized for SARS-CoV-2 serial surveillance testing (screening),^39,40^ we wished to test whether low-sensitivity testing with nasal swabs could provide equivalent performance to high-sensitivity testing with saliva.^15,36,41^

Here we describe results from four transmission events. In the first participant, a 30-39 year old male with diabetes (Fig. 2A), detection occurred first in saliva at low viral load (1.3x10^3^ copies/mL N1 gene, pink circle), while the nasal swab remained negative. RNase P values of the two sample types were similar, indicating the difference was not due to a sampling artifact. Saliva viral load increased gradually over the following two days to about 10^6^ copies/mL and fluctuated around that level for more than a week. Viral load in nasal-swab samples reached the level of LOD of low-sensitivity tests 1.5 days after the first saliva positive samples (pink triangle), indicating that high-sensitivity saliva measurements would have been more effective for early detection of SARS-CoV-2 infection in this participant than low-sensitivity nasal-swab measurements. Of note, this participant became very mildly symptomatic (one mild symptom) several days after his first positive sample. Around this time, nasal swab viral load peaked above 10^9^ copies/mL while saliva viral load remained about 10^6^ copies/mL, indicating that at the peak of the viral load profile, even low-sensitivity testing would provide reliable detection when used with nasal swabs. The number of symptoms increased around day 9 of enrollment, including cough and shortness of breath, while nasal and saliva viral loads declined, potentially indicating increased involvement of the lower respiratory tract. RNase P values remained consistent throughout the collection period for both saliva and nasal swabs, indicating that these changes in viral loads were likely not a sampling artifact but rather reflected the underlying biology of the infection.

The second participant, a 50-59 year old male with no reported comorbidities, showed similar SARS-CoV-2 viral load dynamics (Fig. 2B). The first positive result (pink circle) was in saliva, at low viral load (1.9x10^4^ copies/mL N1 gene). Subsequent saliva viral loads fluctuated around 10^5^-10^6^ copies/mL range. Nasal swab samples remained negative for 1.5 days of positive saliva samples, with the nasal swab viral load exceeding the LOD range of low-sensitivity tests (pink triangle, Fig. 2B) 2.5 days after the initial positive saliva result. Nasal viral load then quickly reached high values (10^9^-10^10^ copies/mL) as the participant developed mild symptoms. This participant remained symptomatic with high nasal viral load through the end of enrollment. Prolonged high viral load may have been related to host factors or viral factors such as infection with a variant of concern, B. 1.429.

The maximum delay in detection between saliva and nasal swab was observed in the third participant, a female child aged 6–11 with no reported comorbidities (see ROI#1 of Fig. 3C). This participant had detectable low load (10^3^-10^4^) of SARS-CoV-2 RNA in saliva for 4 days while nasal swabs remained negative. Nasal swab viral load increased above the LOD of low sensitivity tests only by the end of the 5^th^ day while the participant remained asymptomatic during this entire time period. Two days later the nasal viral load spiked above 10^10^ copies/mL while the participant reported no symptoms and while saliva viral load remained around 10^6^ copies/mL. This slow rise in saliva viral load is distinctive and resembles a similar slow rise in saliva viral load observed in an 18–25-year-old male participant in our previous study.^12^ We do not have sufficient data to establish whether these viral load profiles represent SARS-CoV-2 infection in other children and young adults. If the profile shown in Fig. 2C does describe other individuals, high-sensitivity testing in saliva would represent an opportunity to detect asymptomatic infection in such individuals many days before their viral load spikes to very high, and likely infectious, levels in the nasal swab samples.

The fourth participant, a 50–59-year-old male reporting obesity (Fig. 2D) tested negative for SARS-CoV-2 by a CLIA-lab test 2 days prior to enrollment in the study, but reported symptoms beginning 3 days prior to enrollment. This person would later report more diverse symptoms (including gastrointestinal symptoms) and with higher severity ratings than the other three participants. He tested negative for 2 days after enrollment in our study and his first positive sample was in saliva (pink circle, Fig. 2D). The nasal swab samples did not reach the LOD of low sensitivity tests until 2.5 days after his first positive saliva sample. Remarkably (see ROI#2 in Fig. 2D), saliva viral load spiked to 3.7x10^8^ viral copies/mL (N1 gene target) while SARS-CoV-2 RNA was undetectable in nasal swab even by the high-sensitivity assay used here. This contrast between high and likely infectious viral load in saliva^42^ at the same time point as a negative nasal swab emphasizes the need for careful choice of sampling site and high test sensitivity in the early stages of SARS-CoV-2 infection.

## Conclusions

Our study needs to be interpreted in the context of its limitations. First, all samples were self-collected. Although we provided training to our participants and monitored the quality of their sample collections using RNase P Cq values, our results do not establish whether samples collected by healthcare professionals would provide the same results. Second, most samples were provided either upon waking or just prior to bed, with strict guidance on not eating, drinking, or brushing teeth prior to sample collection. It may be challenging to obtain samples with such level of control during routine screening and testing. Third, we collected samples in a guanidinium-based inactivating and stabilizing buffer that preserves viral RNA. Our results may differ from those obtained with commonly used transport media designed for viral culture, rather than RNA preservation. Finally, our results capture viral load dynamics from a limited number of individuals from one region of one country with limited SARS-CoV-2 diversity. A larger study with individuals of diverse ages, genetic backgrounds, medical conditions, and SARS-CoV-2 lineages would be ideal to provide a more nuanced and representative understanding of viral dynamics in saliva and nasal swab samples.

With these limitations as caveats, we made four conclusions from our study.

First, choosing correct sampling site is critical for early detection of SARS-CoV-2 infection. In our participants, viral RNA appeared first in saliva and second in nasal swab samples. We do not know whether other sampling sites, such as nasopharyngeal swabs or oropharyngeal swabs, would have provided earlier or later detection than saliva. Given our data, early infection viral load dynamics in these additional sampling sites relative to saliva should be investigated.

Second, early in an infection, it is inappropriate to assume that a person is “not infectious” or “has low viral load” based on a measurement from a single sample type such as a nasal swab. Saliva is known to carry infectious virus.^42^ In our study, it was possible to observe a very high (>10^7^-10^8^ copies/mL) viral load in saliva samples while the paired nasal swab was either negative (Fig. 2D, ROI#2) or had low (~10^3^ copies/mL) viral load (Fig. 2D, subsequent day after ROI#2). On those days, it would be dangerous to assume that the person was not infectious based on nasal-swab test results. Quantitative SARS-CoV-2 culture on paired saliva and swab samples would be needed to understand infectiousness during early stages of the SARS-CoV2 infection.

Third, using a diagnostic test with high analytical sensitivity, rather than a test of a particular type, is essential to early detection. Often the test type (e.g., RT-qPCR) is equated to high analytical sensitivity, and some current travel and work guidelines specify a test type (e.g., RT-qPCR) rather than a particular test sensitivity. However, this is a significant oversimplification. FDA testing^11^ demonstrated that sensitivity of RT-qPCR tests ranges from highly sensitive (e.g., 180 NDU/mL for PerkinElmer and 450 NDU/mL for Zymo Research) to substantially less sensitive (e.g., 180,000 NDU/mL for TaqPath COVID-19 Combo Kit and 540,000 NDU/mL for Lyra Direct SARS-CoV-2 Assay). FDA’s NDU/mL is approximately equivalent to the copy/mL scale used in this paper. The low-sensitivity end of this RT-qPCR sensitivity range (corresponding to the higher LOD values) overlaps with the range of low-sensitivity rapid nucleic-acid tests (e.g., 180,000 NDU/mL for Atila BioSystems and 300,000 NDU/mL for Abbott ID NOW tests) and approaches the sensitivity range of antigen tests. Therefore, to achieve early detection, tests with high sensitivity rather than tests of a particular type should be chosen.

Fourth, in our data, when a high-sensitivity test is combined with saliva as a sample type, SARS-CoV-2 infection can be detected up to 4.5 days earlier relative to a low-sensitivity test from a nasal swab. However, during the peak of the infection, in individuals with mild Covid-19 viral load in nasal swab was higher than in saliva, supporting the preferred use of nasal swabs in environments where only low-sensitivity testing is available.

We hope our data and important work by others in this area^13–15,42^ will lead to improved testing strategies for early detection of SARS-CoV-2 infection, to reduce community transmission (especially among unvaccinated populations such as children, or in areas of the world with limited vaccine access), to prevent further development and spread of SARS-CoV-2 variants due to travel, and to enable earlier initiation and thereby more effective therapeutic treatment for COVID-19.

## Methods

### Participant Population

This study is an extension of our previous study examining viral load in saliva.^12^ Both studies were reviewed and approved by the Institutional Review Board of the California Institute of Technology, protocol #20–1026. All participants provided either written informed consent or (for minors ages 6–17) assent accompanied by parental permission, prior to enrollment. Household index cases were eligible for participation if they had recently (within 7 days) been diagnosed with COVID-19 by a CLIA laboratory test. Individuals were ineligible if they were hospitalized or if they were not fluent in either Spanish or English. All participant data were collected and managed using REDCap (Research Electronic Data Capture) on a server hosted at the California Institute of Technology. Demographic and medical information for the four participants described here can be found in Table S1.

### Questionnaires and Symptom Monitoring

Acquisition of participant data was performed as described in our previous study.^12^ Briefly, upon enrollment each participant completed an online questionnaire regarding demographics, health factors, prior COVID-19 tests, COVID-19-like symptoms since February 2020, household infection-control practices, and perceptions of COVID-19 risk. Participants also filled out a post-study questionnaire in which they documented medications taken and their interactions with each household member during their enrollment.

Information on symptoms was collected twice daily in parallel with sample collection. Participants recorded any COVID-19-like symptoms (as defined by the CDC^38^) they were experiencing at the time of sample donation on a symptom-tracking card or on a custom app run on REDCap. Whenever possible, participants indicated the self-reported severity of each symptom. Participants were also given the opportunity to free write-in additional symptoms or symptom details not otherwise listed.

### Collection of Respiratory Specimens

Participants self-collected both their nasal-swab and saliva samples using the Spectrum SDNA-1000 Saliva Collection Kit (Spectrum Solutions LLC, Draper, UT, USA) at home twice per day (after waking up and before going to bed). Saliva samples were collected following the manufacturer’s guidelines. Participants were instructed not to eat, drink, smoke, brush their teeth, use mouthwash, or chew gum for at least 30 min prior to donating. Prior to nasal-swab donation, participants were asked to gently blow their noses to remove debris. Participants were provided with one of the following types of sterile flocked swabs: Nest Oropharyngeal Specimen Collection Swabs (Cat. NST-202003, Stellar Scientific, Baltimore, MD, USA) Puritan HydraFlock Swab (Cat. 25-3000-H E30, Puritan, Guilford, ME, USA) or Copan USA FLOQSwab (Cat. 520CS01, VWR International, Radnor, PA, USA). Participants were instructed to swab each nostril for four complete rotations using the same swab while applying gentle pressure, then to break the tip of the swab into the Spectrum tube and securely screw on the cap. A parent or legal guardian assisted all minors with swab collection and they were instructed to wear a face covering during supervision. Tubes were labelled and packaged by the participants and transported at room temperature by a touch-free medical courier to the California Institute of Technology daily for analysis.

Upon receipt of the samples in the California Institute of Technology laboratory, each sample was inspected for quality. A sample failed quality control if the preservation buffer was not released from the Spectrum SDNA-1000 cap, or if sample tubes were leaking or otherwise unsafe to handle. Samples that failed quality control were not processed. Inactivated samples were stored at 4 °C and were equilibrated to room temperature before being processed with extraction protocols.

### RNA Extraction Protocols

Participant saliva and anterior-nares swab samples were extracted using the KingFisher Flex 96 instrument (ThermoFisher Scientific) with the MagMax Viral Pathogen I Nucleic Acid Isolation kit (Cat. A42352, Applied Biosystems, Waltham, MA, USA) guided by ThermoFisher technical notes for SARS-CoV-2 modification and saliva. Saliva and anterior-nares swabs samples were prepared for purification by transferring 550 μl (for saliva) or 250 μl (for nasal swab) of each sample from its corresponding Spectrum buffer tube into a 1.5 mL lo-bind Eppendorf tube containing 10 μl (for saliva) or 5 μl (for nasal swab) of proteinase K. To maximize recovery of RNA off swabs, prior to transfer, pipet mixing was performed 5–7 times near the swab in the Spectrum tube before aliquoting into an Eppendorf tube. Saliva samples were vortexed for 30 sec in the Eppendorf tube. Samples were incubated at 65 °C for 10 min, then centrifuged at 13,000 x g for 1 min. Aliquots of 400 μl (for saliva) or 200 μl (for nasal swab) were transferred into a KingFisher 96 deep well plate (Cat. 95040450, ThermoFisher Scientific) and processed following KingFisher protocols MVP_400ul_3washes.bdz (for saliva) or MVP_200ul_2washes.bdz (for nasal swab). Ethanol washes were performed with 80% ethanol. Both sample types were eluted into 100 μl of MagMax viral pathogen elution buffer.

### RT-qPCR

Quantification of SARS-CoV-2 was performed as previously described.^12^ Briefly, the CDC^37^ SARS-CoV-2 N1 and N2 primers and probes with an internal control targeting RNase P primer and probe were run in a multiplex RT-qPCR reaction using TaqPath 1-Step Rt-qPCR Mastermix (Cat. A15299, ThermoFisher Scientific). Reactions were run in duplicate on a CFX96 Real Time Instrument (Bio-Rad Laboratories, Hercules, CA, USA).

### RT-ddPCR

Reverse-transcription droplet digital PCR (RT-ddPCR) was performed using the Bio-Rad SARS-CoV-2 Droplet Digital PCR kit (Cat. 12013743 , Bio-Rad). Swab samples were processed following the manufacture’s RUO protocol with 5.5 μl template per 22 μl reaction. A total of 42 participant nasal swab samples were characterized by RT-ddPCR. Modifications were made for saliva samples by reducing the template addition to 2.75 μl per 22 μl reaction. A total of 63 participant saliva samples were characterized by RT-ddPCR. Prior to adding template, samples were diluted into digital range using nuclease-free water. Droplets were created using the QX200 Droplet Generator (Cat #1864002, Bio-Rad), thermocycling performed on Bio-Rad C1000 and detected using the QX200 Droplet Digital PCR system (Cat. 1864001, Bio-Rad). Samples were analyzed with QuantaSoft analysis Pro 1.0.595 software following Bio-Rad’s RUO SARS-CoV-2 guidelines.^43^

### Viral load standard curves

A standard curve was prepared for both the saliva and nasal swab protocols. Samples were prepared with known concentrations (based on the certificate of analysis, COA) of heat-inactivated SARS-CoV-2 particles (Batch 70034991, Cat. NR-52286, BEI Resources, Manassas, VA, USA) in the inactivating buffer from the Spectrum SDNA-1000 Saliva Collection Kit (Spectrum Solutions LLC, Draper, UT, USA). To prepare the samples for the saliva protocol (Fig. S1A) a dilution curve of heat-inactivated SARS-CoV-2 particles (Batch 70034991, Cat no. NR-52286, BEI) in the Spectrum device inactivation buffer at concentrations of 0 copies/mL, 1,000 copies/mL, 2.0 copies/mL, 4,000 copies/mL, 8,000 copies/mL, 16,000 copies/mL, 64,000 copies/mL, 256,000 copies/mL, 1.020.0 copies/mL, and 4,100,000 copies/mL. Samples were made by mixing 620μL of each concentration of the dilution series with 620 μL of healthy pooled human saliva (Cat, 991–05-P, Lee Biosolutions, Maryland Heights, MO, USA). Triplicate extractions were performed according to the saliva RNA extraction protocol. Each extraction was run in triplicate qPCR reactions and single RT-ddPCR reactions.

To prepare the samples for the nasal swab RNA extraction protocol (Fig. S1B) we created dilution curves for each sample type (saliva and nasal swab) and each target (N1 and N2 genes) using heat-inactivated SARS-CoV-2 particles (Batch 70034991, Cat no. NR-52286, BEI) in the Spectrum inactivation buffer. We ran a dilution series of commercial quantified stock (3.75x10^8^ GE/mL) in a 10-fold dilution series from 1x10^6^ to 1x10^4^ copies/mL with finer resolution down to our LOD at 1x10^3^ copies/mL. To each dilution we added 32 μL of healthy human nasal fluid (Cat No 991–13-P-PreC, Lee Biosolutions) to 768 μL of each dilution for a total volume of 800 μL. Triplicate extractions were performed according to the nasal swab RNA extraction protocol (described above). Each extraction was run in triplicate qPCR reactions and single RT-ddPCR reactions.

Equations from the calibration curves are below. These calibration curves are used to convert the Cq values obtained by RT-qPCR to viral load in each participant sample. For saliva, viral load is a calculation of viral copies/mL in the saliva corrected for dilution with the Spectrum buffer. We assumed that participants donate saliva to the fill line, matching the 1:1 dilution in Spectrum buffer recreated when preparing contrived samples for the saliva calibration curve. For nasal swabs, viral load is a calculation of the concentration of viral copies/mL released from the swab into the 1.5 mL of inactivating buffer (which is a similar volume as the 1–3 mL of viral transport media typically used for sample collection). Concentrations higher than 1,000,000 copies/mL could not be characterized due to a limitation of the available stock concentration of commercial inactivated SARS-CoV-2. To validate linear conversion was acceptable at concentrations higher than 1,000,000 copies/mL, we compared RT-ddPCR and RT-qPCR quantification on some participant samples (Figure 1).

(1) $$Saliva\;N1\;gene\;viral\;load\;\left[copies/mL\right]=2^{\left(\left(Cq-46.349\right)/-1.0357\right)}$$

(2) $$Saliva\;N2\;gene\;viral\;load\;\left[copies/mL\right]=2^{\left(\left(Cq-46.374\right)/-1.0759\right)}$$

(3) $$Nasal\;Swab\;N1\;gene\;viral\;load\;\left[copies/mL\right]=2^{\left(\left(Cq-48.221\right)/-1.0643\right)}$$

(4) $$Nasal\;Swab\;N2\;gene\;viral\;load\;\left[copies/mL\right]=2^{\left(\left(Cq-48.330\right)/-1.1044\right)}$$

### Establishment of Limit of Detection

Results of the calibration curve (Fig. S1A,B) demonstrated 3 of 3 replicates detected at 1,000 copies/mL saliva (for saliva) and 1,000 copies/mL buffer (for nasal swabs). For each sample type (saliva, nasal swab), 20 contrived samples with the equivalent of 1,000 copies/mL were prepared as described above, individually extracted as described above, and subjected to RT-qPCR as described above. The LOD for each sample type through the workflow was considered established if a positive result for detection (as defined in the EUA for the CDC RT-qPCR assay) was obtained for ≥ 19 of 20 (≥95%) of replicates at the input concentration (Fig. S2A,B).

Three of three replicate sample extractions included in the calibration curves for both contrived nasal swab samples and contrived saliva samples spiked with heat-inactivated SARS-CoV-2 particles at a concentration of 1,000 copies/mL were detected by RT-qPCR, prompting testing of additional 20 replicates of each sample type spiked at that concentration, individually extracted, and tested by RT-qPCR to establishment of the LOD for our RT-qPCR assay. For both sample types (saliva and nasal swabs), 20 of 20 replicates were positive for SARS-CoV-2 (Fig. S1C,D), establishing 1,000 copies/mL of saliva and 1,000 copies/mL of swab buffer as the high-sensitivity LOD for our RT-qPCR assays.

### Data Analysis

Before we converted Cq values to viral load, we used Cq cutoffs based on the CDC guidelines^37^ to exclude from the viral-load plots any points that were indeterminate or fails, and any samples whose RNase P Cq values ≥40. Because we ran duplicate RT-qPCR reactions, the mean Cq of positive reactions was used for conversion to viral load.

### RNAseq

Saliva and nasal swab samples below N1 Cq of 26 were sent to Chan Zuckerberg Biohub for SARS-CoV-2 viral genome sequencing, a modification of Deng et al. (2020)^44^ as described in Gorzynski et al. (2020).^45^ Sequences were assigned pangolin lineages described by Rambaut et al. (2020)^46^ using Phylogenetic Assignment of Named Global outbreak LINeages software v2.3.2 (github.com/cov-lineages/pangolin).

## Supplementary Material

1
