# Supplementary material for: Quantitative SARS-CoV-2 viral-load curves in paired saliva and nasal swabs inform appropriate respiratory sampling site and analytical test sensitivity required for earliest viral detection
Source: medRxiv. 2021 Aug 26:2021.04.02.21254771. Originally published 2021 Apr 7. Preprint. [Version 2] doi: 10.1101/2021.04.02.21254771 (PMC8043477; doi:10.1101/2021.04.02.21254771)
Supplement: 1 [file NIHPP2021.04.02.21254771V2-supplement-1.pdf]

medRxiv preprint doi: https://doi.org/10.1101/2021.04.02.21254771; this version posted August 26, 2021. The copyright holder for this preprint (which was not certified by peer review) is the author/funder, who has granted medRxiv a license to display the preprint in perpetuity.
It is made available under a CC-BY 4.0 International license .

Supplemental Information for:

Quantitative SARS-CoV-2 viral-load curves in paired saliva and nasal swabs inform appropriate respiratory sampling site and analytical test sensitivity required for earliest viral detection

Emily S. Savela[1#], Alexander Winnett[1#], Anna E. Romano[1#], Michael K. Porter[1], Natasha Shelby[1], Reid Akana[1], Jenny Ji[1], Matthew M. Cooper[1], Noah W. Schlenker[1], Jessica A. Reyes[1], Alyssa M. Carter[1], Jacob T. Barlow[1], Colten Tognazzini[2], Matthew Feaster[2], Ying-Ying Goh[2], Rustem F. Ismagilov[1]*

[1]California Institute of Technology, 1200 E. California Blvd., Pasadena, CA, USA 91125
[2]City of Pasadena Public Health Department, 1845 N. Fair Oaks Ave., Pasadena, CA, USA 91103

*Correspondence to: rustem.admin@caltech.edu
[#] these authors contributed equally

Contents

- Figures S1 – S5
- Table S1
- Supplementary Methods
- Author Contributions

medRxiv preprint doi: https://doi.org/10.1101/2021.04.02.21254771; this version posted August 26, 2021. The copyright holder for this preprint (which was not certified by peer review) is the author/funder, who has granted medRxiv a license to display the preprint in perpetuity.
It is made available under a CC-BY 4.0 International license .

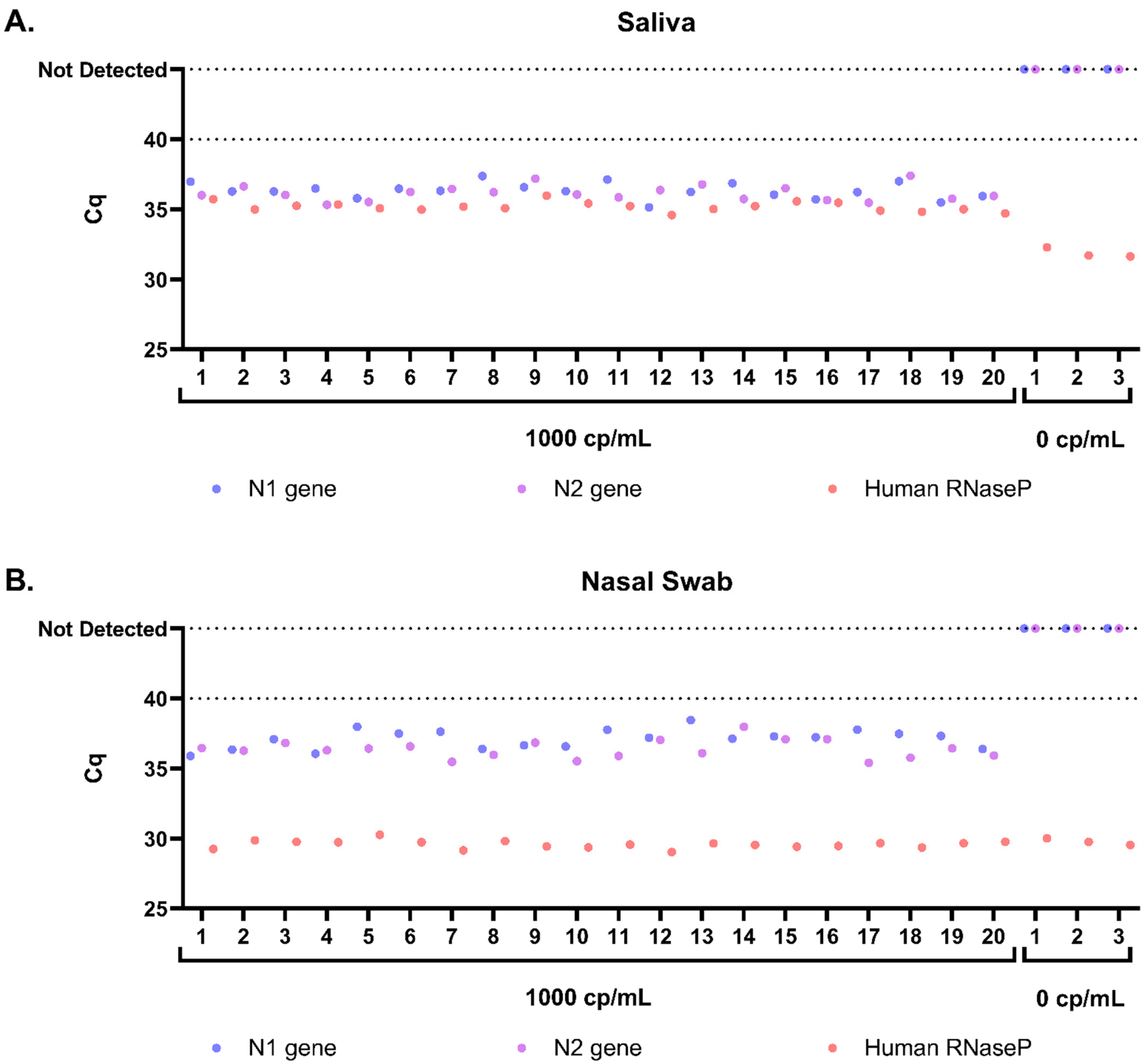

Figure S1. Limit of detection of saliva and nasal-swab RT-qPCR assays used in this study. RT-qPCR quantification cycle (Cq) for SARS-CoV-2 *N1* gene (blue circle), *N2* gene (purple circle), and human *RNase P* gene (orange circle) in 20 replicates of pooled matrix spiked with 1000 copies/mL (cp/mL) heat-inactivated SARS-CoV-2 RNA and 3 replicates of pooled matrix spiked with a buffer blank for saliva (A) and nasal-swab (B) samples. Duplicate RT-qPCR reactions were performed for each extraction replicate and the averages are shown, with the following three exceptions: replicate 9 (saliva), in which the *N1* gene only amplified in 1 of the duplicate runs (*N2* in this run was positive, so per CDC EUA guidelines[87] this run was interpreted as inconclusive), replicate 10 (nasal swab) in which the *N2* gene only amplified in 1 of the duplicate runs (N1 in this run was positive, so this run was interpreted as inconclusive), and replicate 18 (nasal swab) in which the N1 gene only amplified in 1 of the duplicate runs (*N2* in this run was positive, so this run was also interpreted as inconclusive). None of the samples spiked at 1000 copies/mL gave a negative detection result.

medRxiv preprint doi: https://doi.org/10.1101/2021.04.02.21254771; this version posted August 26, 2021. The copyright holder for this preprint (which was not certified by peer review) is the author/funder, who has granted medRxiv a license to display the preprint in perpetuity.
It is made available under a CC-BY 4.0 International license .

A.

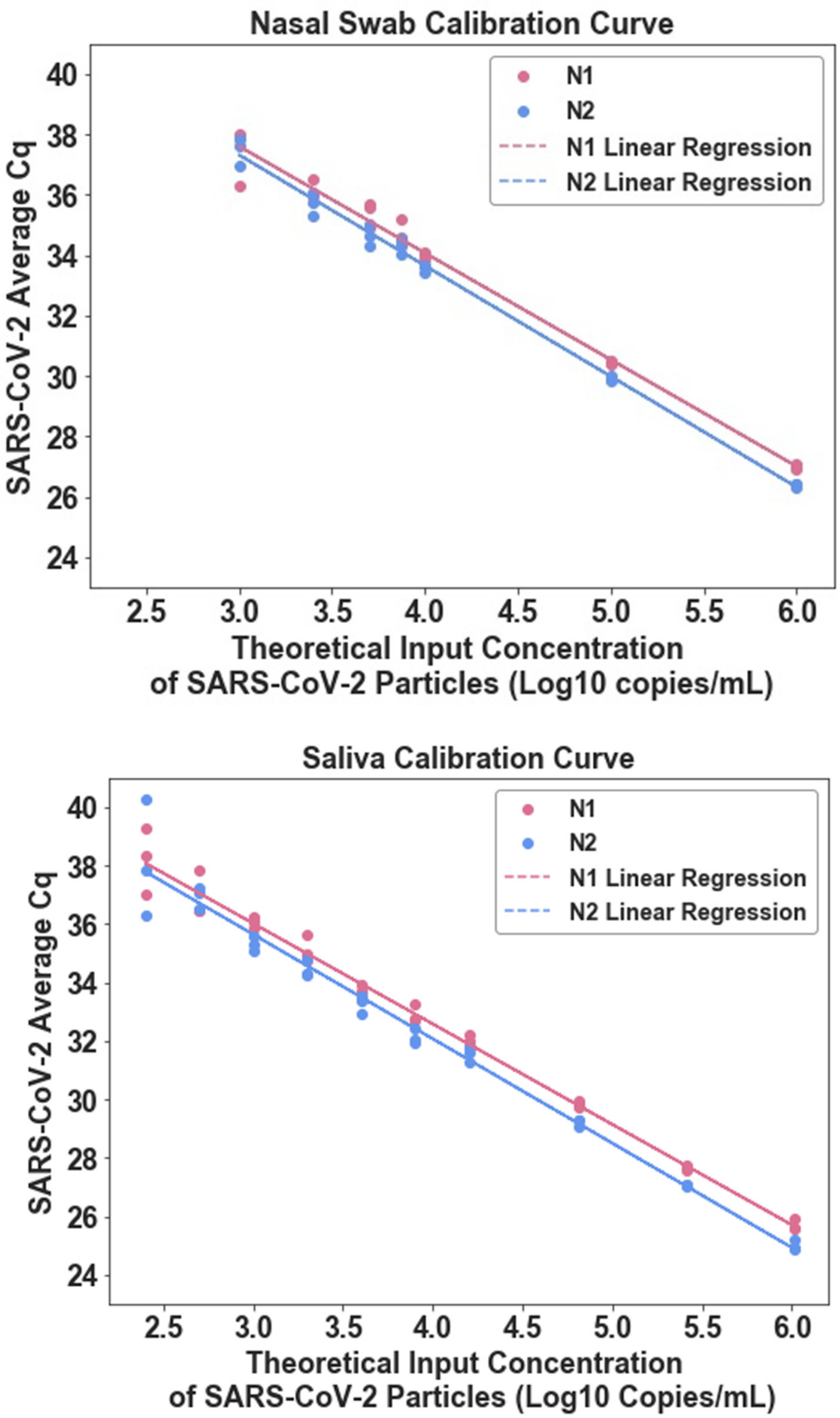

B.

Figure S2. Calibration curve of SARS-CoV-2 inactivated particles to establish viral load conversion equations. Linear regression of RT-qPCR quantification cycle (Cq) for *N1* (red circle) and *N2* (blue circles) genes at known concentrations of inactivated SARS-CoV-2 particles for saliva (A) or nasal swab (B) using this study's collection and laboratory workflows. Triplicate replicates per concentration were performed. Linear regression for *N1* represented by red line and *N2* represented by blue line. Linear regression R^2 values are 0.986 for *N1* in nasal swabs, 0.994 for *N2* in nasal swabs, 0.989 for *N1* in saliva, and 0.979 for *N2* in saliva.

medRxiv preprint doi: https://doi.org/10.1101/2021.04.02.21254771; this version posted August 26, 2021. The copyright holder for this preprint (which was not certified by peer review) is the author/funder, who has granted medRxiv a license to display the preprint in perpetuity.
It is made available under a CC-BY 4.0 International license .

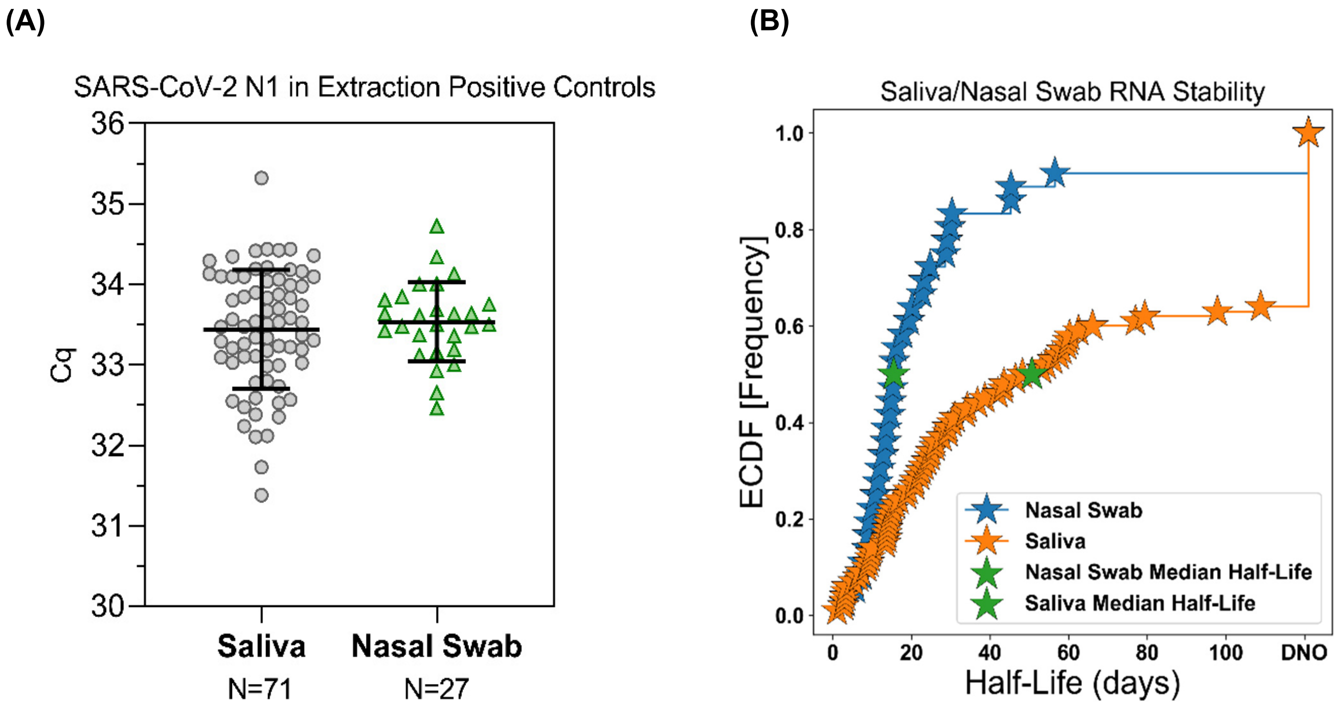

Figure S3. SARS-CoV-2 RNA stability over time in Spectrum SDNA-1000 buffer at 4 °C. (A) Positive extraction control samples from 71 saliva extraction runs and 27 nasal-swab extraction runs are included to show the measurement noise in the quantification workflow. The standard deviation for the positive control measurements was 0.74 Cq for saliva and 0.49 for nasal swab. (B) Empirical cumulative distribution functions (ECDF) for the observed half-life (days) of participant saliva (orange stars) and nasal-swab (blue stars) samples in Spectrum SDNA-1000 buffer stored at 4 °C. Individual samples were extracted at multiple time points. The ECDF represents the summed probability mass function over the Cq half-lives observed. Half-life in this context refers to the time required to observe a 1 Cq increase (representing a 2-fold decrease) in RNA detected by RT-qPCR. The median point is identified with a green star for each sample, at 15.0 days for nasal swabs and 51.0 days for saliva. Of the 110 total participant saliva samples plotted in panel B, 36 samples had no evidence of degradation (DNO) under the time frame measured. Only 3 of the 36 total participant nasal-swab samples plotted in panel B had no evidence of degradation (DNO) under the time frame measured. DNO = degradation not observed, meaning that the difference in extraction Cq values of the same sample at multiple time points was within 1 standard deviation observed in replicate extraction positive controls for the respective sample type, as shown in panel A.

medRxiv preprint doi: https://doi.org/10.1101/2021.04.02.21254771; this version posted August 26, 2021. The copyright holder for this preprint (which was not certified by peer review) is the author/funder, who has granted medRxiv a license to display the preprint in perpetuity.
It is made available under a CC-BY 4.0 International license .

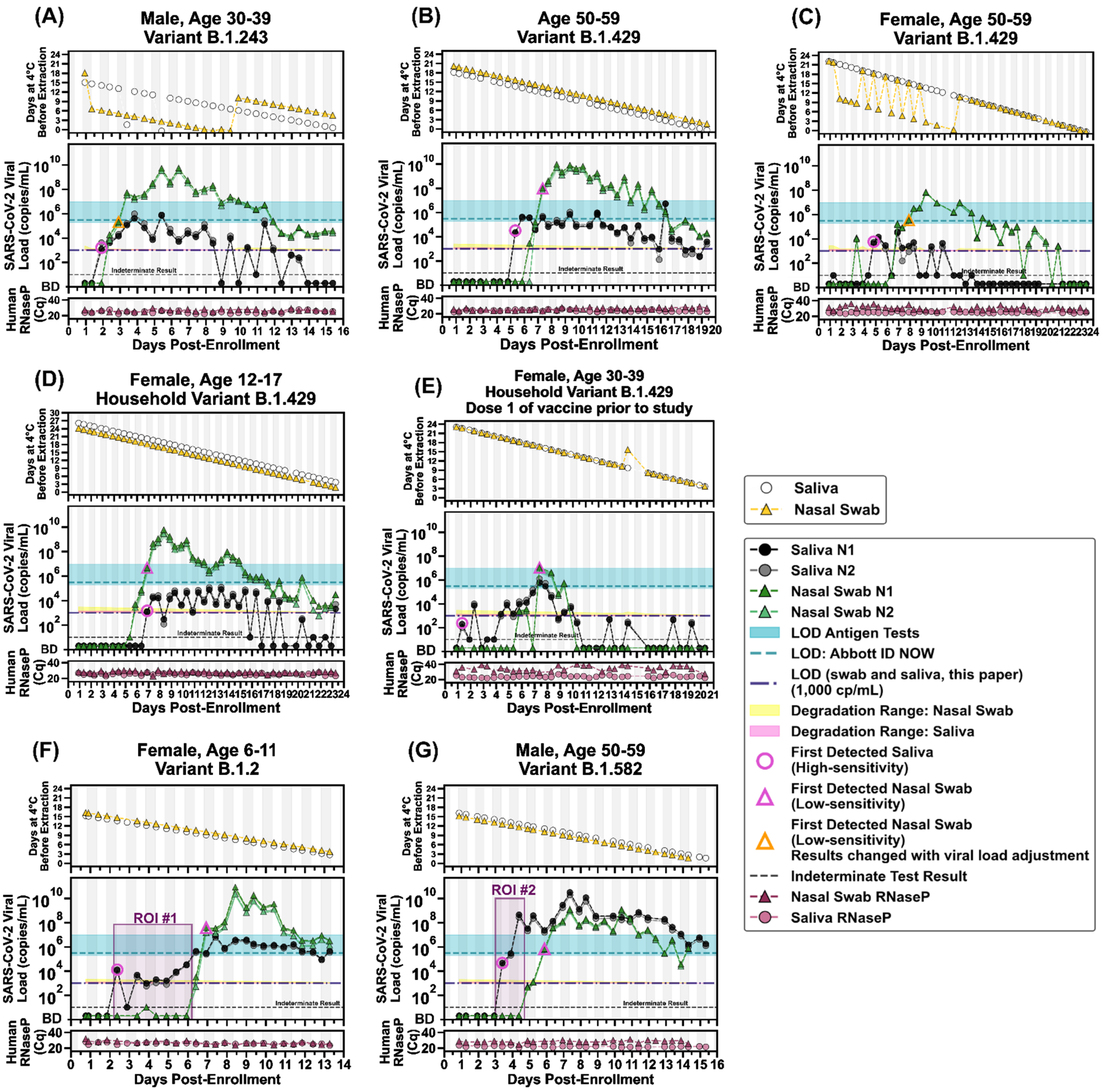

Figure S4. Predicted impact of SARS-CoV-2 RNA stability on viral loads shown in Fig. 2. (A-G) The time [days] of sample storage at 4 °C between sample collection and RNA extraction is shown in the topmost panels. Open circles represent saliva samples and yellow triangles represent the nasal swabs. Viral load (black and grey circles) calculations are corrected for the median half-life (1 Cq decrease in RNA detected by RT-qPCR) of each sample type and the duration of storage at 4 °C before quantification (15 days for 2-fold decrease in detected RNA in nasal swabs and 51 days for 2-fold decreased in detected RNA in saliva). The degradation ranges, represented by a shaded yellow (nasal swab) or pink (saliva) region to represent how a measured value of 1,000 copies/mL may have degraded from concentrations in this range. As in Fig. 2, ND = not detected for Cqs ≥40 (see Methods for details). The limit of detection (LOD) of the saliva and nasal-swab assays used here (1,000 cp/mL) is indicated with the purple dashed line; the LOD of the Abbott ID NOW (300,000 NDU/mL[17]) is indicated by the horizontal green dashed line; the range of LODs of antigen tests (horizontal green bar) are shown for reference (data are from Table S2 in ref. [19]). A diagnostic test does not provide reliable detection for samples

medRxiv preprint doi: https://doi.org/10.1101/2021.04.02.21254771; this version posted August 26, 2021. The copyright holder for this preprint (which was not certified by peer review) is the author/funder, who has granted medRxiv a license to display the preprint in perpetuity. It is made available under a CC-BY 4.0 International license .

with viral loads below its LOD. For each participant, the first detected saliva point is emphasized with a pink circle and their first nasal-swab point above the LOD of the ID NOW is emphasized with a pink triangle. Vertical shading in grey indicates nighttime (8pm – 8am). Internal control of *RNase P* gene Cqs from the CDC primer set are provided for each sample to compare self-sampling consistency and sample integrity (failed samples, where *RNase P* Cq ≥40, are not plotted). Samples with an indeterminate result by the CDC RT-qPCR assay are shown along the horizontal black dashed line. Participant gender, age range, and SARS-CoV-2 variant is given in each panel's title. Two regions of interest (ROI) are indicated by purple-shaded rectangles and discussed in the main text. For the two points that change interpretation with the viral load adjustment, orange triangles show which new data points become the first nasal-swab point in range of low-sensitivity tests.

medRxiv preprint doi: https://doi.org/10.1101/2021.04.02.21254771; this version posted August 26, 2021. The copyright holder for this preprint (which was not certified by peer review) is the author/funder, who has granted medRxiv a license to display the preprint in perpetuity.
It is made available under a CC-BY 4.0 International license .

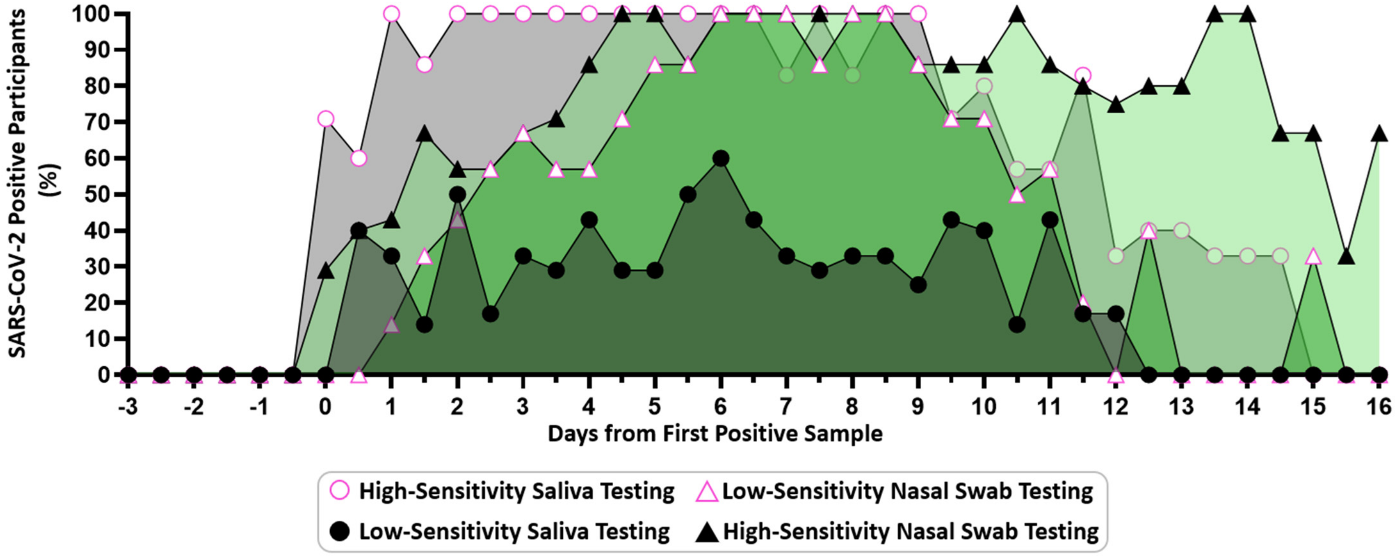

Figure S5. Participants positive by each sample type and test sensitivity at each timepoint from the incidence of infection. Infection time courses were aligned to the first positive result from our high-sensitivity (LOD of ≤1e3 copies/mL) testing for either sample type. Using the individual SARS-CoV-2 *N1* viral load values for each participant individually shown in Figure 2, we show the percentage of participants who were detected by our high-sensitivity saliva (black circle with pink border) or nasal swab (green triangle) assay (LOD ≤1e3 copies/mL) or could be inferred to be detectable by a low-sensitivity nasal swab (green triangle with pink border) or saliva (black circle) test (with an LOD of 1.9e5 copies/mL) at a given timepoint. Positivity by the high-sensitivity saliva test and low-sensitivity nasal swab test is also shown in Fig. 3 panel A.

medRxiv preprint doi: https://doi.org/10.1101/2021.04.02.21254771; this version posted August 26, 2021. The copyright holder for this preprint (which was not certified by peer review) is the author/funder, who has granted medRxiv a license to display the preprint in perpetuity.
It is made available under a CC-BY 4.0 International license .

Table S1. Study participant demographic data. Figure 2 shows viral loads and symptoms data for the seven participants for whom we observed transmission during their enrollment in the study.

	**Age Range (Years)**	**Sex**	**Race; Ethnicity**	**Reported Medical Conditions Associated with Increased Risk of Severe COVID-19[88]**
Fig. 2A, Fig. S4A	30-39	Male	Other; Mexican/Mexican-American/Chicano (Salvadoran)	Diabetes
Fig. 2B, Fig. S4B	50-59	Male	Do not wish to respond; Mexican/Mexican-American/Chicano	None
Fig. 2C, Fig. S4C	50-59	Female	White; Mexican/Mexican-American/Chicano (Spanish-American from Spain)	None
Fig. 2D, Fig. S4D	12-17	Female	White; Mexican/Mexican-American/Chicano	None
Fig. 2E, Fig. S4E	30-39	Female	White; Mexican/Mexican-American/Chicano	None
Fig. 2F, Fig. S4F	6-11	Female	White; Non-Hispanic	None
Fig. 2G, Fig. S4G	50-59	Male	American Indian or Alaskan Native, White; Other Hispanic, Latinx or Spanish origin	Obesity

medRxiv preprint doi: https://doi.org/10.1101/2021.04.02.21254771; this version posted August 26, 2021. The copyright holder for this preprint (which was not certified by peer review) is the author/funder, who has granted medRxiv a license to display the preprint in perpetuity.
It is made available under a CC-BY 4.0 International license .

Supplementary Methods

Participant Population

This study is an extension of our previous study examining viral load in saliva.[19] Both studies were reviewed and approved by the Institutional Review Board of the California Institute of Technology, protocol #20-1026. All participants provided either written informed consent or (for minors ages 6-17) assent accompanied by parental permission, prior to enrollment. Household index cases were eligible for participation if they had recently (within 7 days) been diagnosed with COVID-19 by a CLIA laboratory test. Individuals were ineligible if they were hospitalized or if they were not fluent in either Spanish or English. All participant data were collected and managed using REDCap (Research Electronic Data Capture) on a server hosted at the California Institute of Technology. Demographic and medical information for the seven participants described here can be found in Table S1.

Questionnaires and Symptom Monitoring

Acquisition of participant data was performed as described in our previous study.[19] Briefly, upon enrollment each participant completed an online questionnaire regarding demographics, health factors, prior COVID-19 tests, COVID-19-like symptoms since February 2020, household infection-control practices, and perceptions of COVID-19 risk. Participants also filled out a post-study questionnaire in which they documented medications taken and their interactions with each household member during their enrollment.

Information on symptoms was collected twice daily in parallel with sample collection. Participants recorded any COVID-19-like symptoms (as defined by the CDC[83]) they were experiencing at the time of sample donation on a symptom-tracking card or on a custom app run on REDCap. Whenever possible, participants indicated the self-reported severity of each symptom. Participants were also given the opportunity to write-in additional symptoms or symptom details not otherwise listed.

Collection of Respiratory Specimens

Participants self-collected both their nasal-swab and saliva samples using the Spectrum SDNA-1000 Saliva Collection Kit (Spectrum Solutions LLC, Draper, UT, USA), which contains 1.5 mL of liquid buffer, at home twice per day (after waking up and before going to bed), per manufacturer guidelines. Of note, at the time of this writing, Spectrum devices are not approved for the collection of nasal-swab samples. Participants self-collected nasal-swab (1 swab) and saliva (~1.5mL) samples in the Spectrum SDNA-1000 Saliva Collection Kit (Spectrum Solutions LLC, Draper, UT, USA), which contains 1.5mL of liquid buffer, at home twice per day (after waking up and before going to bed), per manufacturer's guidelines. Of note, at the time of this writing, Spectrum devices are not approved for the collection of nasal-swab samples.

Participants were instructed not to eat, drink, smoke, brush their teeth, use mouthwash, or chew gum for at least 30 min prior to donating. Prior to nasal-swab donation, participants were asked to gently blow their noses to remove debris. Participants were provided with one of the following types of sterile flocked swabs: Nest Oropharyngeal Specimen Collection Swabs (Cat. NST-202003, Stellar Scientific, Baltimore, MD, USA) Puritan HydraFlock Swab (Cat. 25-3000-H E30, Puritan, Guilford, ME, USA) or Copan USA FLOQSwab (Cat. 520CS01, VWR International, Radnor, PA, USA). Participants were instructed to swab each nostril for four complete rotations using the same swab while applying gentle pressure, then to break the tip of the swab into the Spectrum tube and securely screw on the cap. A parent or legal guardian assisted all minors with swab collection and they were instructed to wear a face covering during supervision. Tubes were labeled and packaged by the participants and transported at room temperature by a touch-free medical courier to the California Institute of Technology daily for analysis.

medRxiv preprint doi: https://doi.org/10.1101/2021.04.02.21254771; this version posted August 26, 2021. The copyright holder for this preprint (which was not certified by peer review) is the author/funder, who has granted medRxiv a license to display the preprint in perpetuity.
It is made available under a CC-BY 4.0 International license .

Upon receipt of the samples in the California Institute of Technology laboratory, each sample was inspected for quality. A sample failed quality control if the preservation buffer was not released from the Spectrum SDNA-1000 cap, or if sample tubes were leaking or otherwise unsafe to handle. Samples that failed quality control were not processed. Inactivated samples were stored at 4 °C and were equilibrated to room temperature before being processed with extraction protocols.

RNA Extraction Protocols

Participant saliva and anterior-nares swab samples were extracted using the KingFisher Flex 96 instrument (ThermoFisher Scientific) with the MagMax Viral Pathogen I Nucleic Acid Isolation kit (Cat. A42352, Applied Biosystems, Waltham, MA, USA) guided by ThermoFisher technical notes for SARS-CoV-2 modification and saliva. Each extraction batch, depending on the sample type being extracted, contained a contrived SARS-CoV-2 negative control sample containing either 225 µL of Spectrum buffer mixed with 225 µL of commercial pooled human saliva (Lee Bio 991-05-P-PreC) or 240 µL of Spectrum buffer with 10 µL of pooled commercial nasal fluid (Lee Bio 991-13-P-PreC); a contrived SARS-CoV-2 positive control sample was also included in each extraction batch, with the formulations above, but with the Spectrum buffer spiked with 7,500 genomic copy equivalents/mL of heat-inactivated SARS-CoV-2 particles (BEI NR-52286).

Saliva and anterior-nares swab samples were prepared for purification by transferring 550 µl (for saliva) or 250 µl (for nasal swab) of each sample from its corresponding Spectrum buffer tube into a 1.5 mL lo-bind Eppendorf tube containing 10 µl (for saliva) or 5 µl (for nasal swab) of proteinase K. To maximize recovery of RNA off swabs, prior to transfer, pipet mixing was performed 5-7 times near the swab in the Spectrum tube before aliquoting into an Eppendorf tube. Saliva samples were vortexed for 30 sec in the Eppendorf tube. Samples were incubated at 65 °C for 10 min, then centrifuged at 13,000 x g for 1 min. Aliquots of 400 µl (for saliva) or 200 µl (for nasal swab) were transferred into a KingFisher 96 deep well plate (Cat. 95040450, ThermoFisher Scientific) and processed following KingFisher protocols MVP_400ul_3washes.bdz (for saliva) or MVP_200ul_2washes.bdz (for nasal swab). Ethanol washes were performed with 80% ethanol. Both sample types were eluted into 100 µl of MagMax viral pathogen elution buffer.

RT-qPCR

Quantification of SARS-CoV-2 was performed as previously described.[19] Briefly, the CDC[66] SARS-CoV-2 *N1* and *N2* gene primers and probes with an internal control targeting *RNase P* gene primer and probe were run in a multiplex RT-qPCR reaction using TaqPath 1-Step Rt-qPCR Mastermix (Cat. A15299, ThermoFisher Scientific). Reactions were run in duplicate on a CFX96 Real-Time Instrument (Bio-Rad Laboratories, Hercules, CA, USA).

RT-ddPCR

Reverse-transcription droplet digital PCR (RT-ddPCR) was performed using the Bio-Rad SARS-CoV-2 Droplet Digital PCR kit (Cat. 12013743, Bio-Rad). Swab samples were processed following the manufacturer's RUO protocol with 5.5 µl template per 22 µl reaction. A total of 42 participant nasal-swab samples were characterized by RT-ddPCR. Modifications were made for saliva samples by reducing the template addition to 2.75 µl per 22 µl reaction. A total of 63 participant saliva samples were characterized by RT-ddPCR. Prior to adding template, samples were diluted into digital range using nuclease-free water. Droplets were created using the QX200 Droplet Generator (Cat #1864002, Bio-Rad), thermocycling performed on Bio-Rad C1000 and detected using the QX200 Droplet Digital PCR system (Cat. 1864001, Bio-Rad). Samples were analyzed with QuantaSoft analysis Pro 1.0.595 software following Bio-Rad's RUO SARS-CoV-2 guidelines.[67]

medRxiv preprint doi: https://doi.org/10.1101/2021.04.02.21254771; this version posted August 26, 2021. The copyright holder for this preprint (which was not certified by peer review) is the author/funder, who has granted medRxiv a license to display the preprint in perpetuity.
It is made available under a CC-BY 4.0 International license .

Viral Load Standard Curves

A standard curve was prepared for both the saliva and nasal-swab protocols. Samples were prepared with known concentrations (based on the certificate of analysis, COA) of heat-inactivated SARS-CoV-2 particles (Batch 70034991, Cat. NR-52286, BEI Resources, Manassas, VA, USA) in the inactivating buffer from the Spectrum SDNA-1000 Saliva Collection Kit (Spectrum Solutions LLC, Draper, UT, USA). To prepare the samples for the saliva protocol (Fig. S2A) a dilution curve of heat-inactivated SARS-CoV-2 particles (Batch 70034991, Cat no. NR-52286, BEI) in the Spectrum device inactivation buffer at concentrations of 0 copies/mL, 1,000 copies/mL, 2,000 copies/mL, 4,000 copies/mL, 8,000 copies/mL, 16,000 copies/mL, 64,000 copies/mL, 256,000 copies/mL, 1,020,000 copies/mL, and 4,100,000 copies/mL. Samples were made by mixing 620 μL of each concentration of the dilution series with 620 μL of healthy pooled human saliva (Cat, 991-05-P, Lee Biosolutions, Maryland Heights, MO, USA). Triplicate extractions were performed according to the saliva RNA extraction protocol. Each extraction was run in triplicate qPCR reactions and single RT-ddPCR reactions.

To prepare the samples for the nasal-swab RNA extraction protocol (Fig. S2B) we created dilution curves for each sample type (saliva and nasal swab) and each target (*N1* and *N2* genes) using heat-inactivated SARS-CoV-2 particles (Batch 70034991, Cat no. NR-52286, BEI) in the Spectrum inactivation buffer. We ran a dilution series of commercial quantified stock (3.75×10^8 GE/mL) in a 10-fold dilution series from 1×10^6 to 1×10^4 copies/mL with finer resolution down to our LOD at 1×10^3 copies/mL. To each dilution we added 32 μL of healthy human nasal fluid (Cat No 991-13-P-PreC, Lee Biosolutions) to 768 μL of each dilution for a total volume of 800 μL. Triplicate extractions were performed according to the nasal-swab RNA extraction protocol (described above). Each extraction was run in triplicate qPCR reactions and single RT-ddPCR reactions.

Equations from the calibration curves are below. These calibration curves are used to convert the Cq values obtained by RT-qPCR to viral load in each participant sample. For saliva, viral load is a calculation of viral copies/mL in the saliva corrected for dilution with the Spectrum buffer. We assumed that participants donate saliva to the fill line, matching the1:1 dilution in Spectrum buffer recreated when preparing contrived samples for the saliva calibration curve. For nasal swabs, viral load is a calculation of the concentration of viral copies/mL released from the swab into the 1.5 mL of inactivating buffer (which is a similar volume as the 1-3 mL of viral transport media typically used for sample collection). Concentrations higher than 1,000,000 copies/mL could not be characterized due to a limitation of the available stock concentration of commercial inactivated SARS-CoV-2. To validate linear conversion was acceptable at concentrations higher than 1,000,000 copies/mL, we compared RT-ddPCR and RT-qPCR quantification on some participant samples (Fig. 1).

$$(1)\ Saliva\ N1\ gene\ viral\ load\ [copies/mL] = 2^{((Cq-46.349)/-1.0357)}$$

$$(2)\ Saliva\ N2\ gene\ viral\ load\ [copies/mL] = 2^{((Cq-46.374)/-1.0759)}$$

$$(3)\ Nasal\ Swab\ N1\ gene\ viral\ load\ [copies/mL] = 2^{((Cq-48.221)/-1.0643)}$$

$$(4)\ Nasal\ Swab\ N2\ gene\ viral\ load\ [copies/mL] = 2^{((Cq-48.330)/-1.1044)}$$

Establishment of Limit of Detection

Results of the calibration curve (Fig. S2 A,B) demonstrated 3 of 3 replicates detected at 1,000 copies/mL saliva (for saliva) and 1,000 copies/mL buffer (for nasal swabs). For each sample type (saliva, nasal swab), 20 contrived samples with the equivalent of 1,000 copies/mL were prepared as described above, individually extracted as

medRxiv preprint doi: https://doi.org/10.1101/2021.04.02.21254771; this version posted August 26, 2021. The copyright holder for this preprint (which was not certified by peer review) is the author/funder, who has granted medRxiv a license to display the preprint in perpetuity.
It is made available under a CC-BY 4.0 International license .

described above, and subjected to RT-qPCR as described above. The LOD for each sample type through the workflow was considered established if a positive result for detection (as defined in the EUA for the CDC RT-qPCR assay) was obtained for ≥ 19 of 20 (≥95%) of replicates at the input concentration (Fig. S1 A,B).

Three of three replicate sample extractions included in the calibration curves for both contrived nasal-swab samples and contrived saliva samples spiked with heat-inactivated SARS-CoV-2 particles at a concentration of 1,000 copies/mL were detected by RT-qPCR, prompting testing of additional 20 replicates of each sample type spiked at that concentration, individually extracted, and tested by RT-qPCR to establishment of the LOD for our RT-qPCR assay. For both sample types (saliva and nasal swabs), 20 of 20 replicates were positive for SARS-CoV-2 (Fig. S1 A,B), establishing 1,000 copies/mL of saliva and 1,000 copies/mL of swab buffer as the high-sensitivity LOD for our RT-qPCR assays.

Data Analysis

Before we converted Cq values to viral load, we used Cq cutoffs based on the CDC guidelines[66] to exclude from the viral-load plots any points that were indeterminate or fails, and any samples whose RNase P Cq values ≥40. Because we ran duplicate RT-qPCR reactions, the mean Cq of positive reactions was used for conversion to viral load.

RNAseq

Saliva and nasal-swab samples below *N1* Cq of 26 were sent to Chan Zuckerberg Biohub for SARS-CoV-2 viral genome sequencing, a modification of Deng et al. (2020)[84] as described in Gorzynski et al. (2020).[85] Sequences were assigned pangolin lineages described by Rambaut *et al*. (2020)[86] using Phylogenetic Assignment of Named Global outbreak LINeages software v2.3.2 (github.com/cov-lineages/pangolin). Consequences viral genomes were submitted to GISAID by Chan Zuckerberg Biohub, see data availability section for accession id details.

SARS-CoV-2 RNA Stability at 4 °C

As described above, each extraction batch included a contrived sample spiked with SARS-CoV-2 heat-inactivated particles. For all available saliva or nasal-swab extraction batches, the Cq value of the SARS-CoV-2 *N1* gene in the contrived SARS-CoV-2 positive extraction control was collected. The standard deviation of these measurements was calculated and used to establish a threshold for expected noise between repeat extractions of the same sample. To assess samples for evidence of SARS-CoV-2 RNA degradation, any participant sample that had more than one extraction replicate performed were analyzed. Samples where the difference in Cq values between the extractions was less than the threshold of expected noise between replicate extractions were defined as degradation not observed, (DNO). For samples where the difference was above this threshold, the time for 1 Cq increase (2-fold decrease) in RNA detected by RT-qPCR is described by the term half-life, which was calculated according to Equation 5, below:

$$(5)\ t_{1/2} = \frac{-ln2}{k}$$

Where "k" is defined as the slope of the linear regression of the natural logarithm of the viral load vs. extraction date (relative to sample collection date). Empirical cumulative distribution functions (ECDF) were calculated using the statsmodels Python package. Confidence intervals were constructed using 2500 bootstrap trials. The median over the entire dataset (saliva or swab) was used as a point estimate of RNA half-life.

Calculations that predict the impact of storage time at 4 °C and RNA stability on viral load are calculated according to Equation 6, below.

medRxiv preprint doi: https://doi.org/10.1101/2021.04.02.21254771; this version posted August 26, 2021. The copyright holder for this preprint (which was not certified by peer review) is the author/funder, who has granted medRxiv a license to display the preprint in perpetuity.
It is made available under a CC-BY 4.0 International license .

$$(6)\ y_{adj} = y_{deg} 2^{\frac{\Delta t}{t_{1/2}}}$$

Where y_{adj} is defined as the adjusted viral load, y_{deg} is defined as the viral load before adjustment for degradation (as calculated by Equations 1-4), and $t_{1/2}$ is defined as the RNA half-life, shown in Equation 5.

medRxiv preprint doi: https://doi.org/10.1101/2021.04.02.21254771; this version posted August 26, 2021. The copyright holder for this preprint (which was not certified by peer review) is the author/funder, who has granted medRxiv a license to display the preprint in perpetuity.
It is made available under a CC-BY 4.0 International license .

Author Contributions

Listed alphabetically by last name

Reid Akana (RA): Assisted in literature analysis with ES, MKP, AW, MC; collaborated with AW in creating digital participant symptom surveys; assisted with data quality control/curation with JJ, NWS, NS; collaborated with ES, JJ to write data analysis/visualization code; created current laboratory information management system (LIMS) for specimen logging and tracking. Creation of iOS application for sample logging/tracking. Configured a SQL database for data storage. Created an Apache server and websites to view study data. Configured FTPS server to catalog PCR data. Wrote a Python package to access study data. Worked with ES, AW, AR to implement logic that prioritized specimen extraction order. Collaborated with ES, MKP, AW, AR in analyzing RNA stability. Created supplementary figures 3B and 3C.

Jacob T. Barlow (JTB): Created initial specimen tracking database to aid in specimen logging and tracking. Maintenance of database and implementation of corrections. Feedback on manuscript draft.

Alyssa M. Carter (AMC): Received and logged specimens, and performed sample QC. Prepared reagents for and assisted with RNA extractions. Performed RT-qPCR and analyzed RT-qPCR data for both time series and screening experiments. Performed some of the initial experiments that assessed RNA stability in nasal swab samples

Matthew M. Cooper (MMC): Collaborated with AW, MF, NS, YG, RFI, on study design and recruitment strategies. Co-wrote initial IRB protocol and informed consent with AW and NS; assisted in the writing of the enrollment questionnaire; developed laboratory sample processing workflow for saliva with AW and AER; performed sample processing on subset of samples; funding acquisition; collaborated with AR to write data processing/visualization code for observing household transmission events for active study participants. Contributor to the design of the calibration curve for saliva LOD experiments.

Matthew Feaster (MF): Co-investigator; collaborated with AW, MMC, NS, YG, RFI on study design and recruitment strategies; provided guidance and expertise on SARS-CoV-2 epidemiology and local trends.

Ying-Ying Goh (YYG): Co-investigator; collaborated with AW, MMC, NS, MF, RFI on study design and recruitment strategies; provided guidance and expertise on SARS-CoV-2 epidemiology and local trends.

Rustem F. Ismagilov (RFI): Co-investigator; collaborated with AW, MMC, NS, MF, YYG on study design and recruitment strategies; provided leadership, technical guidance, oversight, and was responsible for obtaining funding for the study.

Jenny Ji (JJ): Contributed to study design and study organization and implementation with NS and JAR; co-wrote enrollment questionnaire with NS and AW. Major contributor to curation of participant symptom data. Provided quality control of participant data with RA, NS, NWS. Major contributor to the symptom data analysis and visualization shown in Fig. 2.

Michael K. Porter (MKP): Performed specimen logging and QC, RNA extractions, RT-qPCR, data processing. Performed data acquisition and analysis for and made Figure S1 with AW. Prepared participant sample collection materials and helped with supplies acquisition. Assisted in literature analysis with ES, RA, AW.

Jessica A. Reyes (JAR): Study coordinator; collaborated with NS, AW, NWS, and RFI on recruitment strategies, translated study materials into Spanish, co-wrote informational sheets with AW and NS; created instructional videos for participants; enrolled and maintained study participants with NS and NWS.

Anna E. Romano (AER): Developed laboratory swab sample processing workflow with ES. Optimized extraction and ddPCR protocols working with vendor scientists. Created budgets and managed, planned, and purchased reagents and supplies; developed and validated method for RT-qPCR and RT-digital droplet PCR analysis for saliva & swab samples with MMC, and AW. Performed specimen logging and QC, RNA extractions, RT-qPCR

medRxiv preprint doi: https://doi.org/10.1101/2021.04.02.21254771; this version posted August 26, 2021. The copyright holder for this preprint (which was not certified by peer review) is the author/funder, who has granted medRxiv a license to display the preprint in perpetuity.
It is made available under a CC-BY 4.0 International license .

and RT-digital droplet PCR; Design of saliva calibration curve experiment. Analyzed ddPCR data for participant and calibration curve data included in Figure 1. Interpretation of sequence data with AW. Prepared Figure 1 and SI Figure S2 with ES and AW; Collaborated with ES and RA to generate and curate data for RNA stability analysis. Managing logistics for the expansion of the BSL-2+ lab space with ESS. Edited manuscript.

Emily S. Savela (ESS): Coordinated the laboratory team and division of lab work, coordinated lab schedules to ensure completion of time-sensitive analyses of participant samples while complying with COVID-19 lab occupancy restrictions and biosafety requirements. Performed initial nasal-swab workflow validation experiments with AER. Major contributor to workflow validation, methods, biosafety SOPs, and sample storage. Developed a plan for, and executed, the long term sample storage for efficient, safe, storage. Performed specimen logging and QC, RNA extractions, RT-qPCR, data processing, and conducted biosafety training. Performed the data curation and data analysis for Figure 2. Made Figure 2. Minor contributor to symptoms data analysis and visualization with JJ and RA for Figure 2. Experimental design and RNA extractions of the samples, to Figure 1A and minor contributor to Figure S2A with AER. Managing logistics for the expansion of the BSL-2+ lab space with AER and biohazardous waste pickups. Collaborated with ES, RA, and AW to generate data for and curated data set to assess viral RNA stability (Fig. S3). Prepared Fig. S4. Co-wrote the manuscript. Verified the underlying data with AW.

Noah W. Schlenker (NWS): Study coordinator; collaborated with NS, AW, JAR, and RFI on recruitment strategies; enrolled and maintained study participants with NS and JAR; study-data quality control, curation and archiving with RA, JJ, and NS.

Natasha Shelby (NS): Study administrator; collaborated with AW, MMC, RFI, YG, MF on initial study design and recruitment strategies; co-wrote IRB protocol and informed consent with AW and MMC; co-wrote enrollment questionnaire with AW and JJ; co-wrote participant informational sheets with AW and JAR; enrolled and maintained study participants with JAR and NWS; study-data quality control, curation and archiving with RA, JJ, and NWS; reagents and supplies acquisition; assembled Table S1; managed citations and reference library; co-wrote and edited the manuscript.

Colten Tognazzini (CT): Coordinated the recruitment efforts at PPHD with case investigators and contact tracers; provided guidance and expertise on SARS-CoV-2 epidemiology and local trends.

Alexander Winnett (AW): Collaborated with MMC, NS, RFI, YG, MF on initial study design and recruitment strategies; co-wrote IRB protocol and informed consent with MMC and NS; co-wrote enrollment questionnaire with NS and JJ; co-wrote participant informational sheets with NS and JAR and digital survey; developed and validated methods for saliva and nasal-swab sample collection; developed and validated methods for RT-qPCR and RT-digital droplet PCR analysis for saliva and swab samples with AER, ESS, MMC; reagents and supplies acquisition; funding acquisition; developed laboratory sample processing workflow with AER, ESS, and MMC; performed specimen logging and QC, nucleic acid extraction, RT-qPCR, data processing – including experimental data generation for saliva calibration curve (Fig. 1, Fig. S2) designed with MMC and AER, establishment of nasal swab limit of detection (Fig. S1), and viral load timeseries data (Fig. 2) with ESS, AER, MKP, and AMC; interpreted sequencing data with AER; analyzed viral load timeseries data to visualize trends (Fig. 3, Fig. S4) with ESS; generated, analyzed and visualized data to assess degradation of viral RNA in saliva and nasal swab samples with RA, ESS, and AER (Fig. S3); literature analysis with RA, ESS, and MKP; co-wrote sections of the manuscript outlined by ESS and RFI, edited the manuscript. Verified the underlying data with ESS.
