# Supplementary material for: SARS-CoV-2 is detectable using sensitive RNA saliva testing days before viral load reaches detection range of low-sensitivity nasal swab tests
Source: medRxiv. 2021 Apr 7:2021.04.02.21254771. Preprint. [Version 1] doi: 10.1101/2021.04.02.21254771 (PMC8043477; doi:10.1101/2021.04.02.21254771)
Supplement: Supplementary file 1 [file NIHPP2021.04.02.21254771-supplement-1.pdf]

medRxiv preprint doi: https://doi.org/10.1101/2021.04.02.21254771; this version posted April 7, 2021. The copyright holder for this preprint (which was not certified by peer review) is the author/funder, who has granted medRxiv a license to display the preprint in perpetuity.
It is made available under a CC-BY 4.0 International license .

Supplemental Information for:

SARS-CoV-2 is detectable using sensitive RNA saliva testing days before viral load reaches detection range of low-sensitivity nasal swab tests

Emily S. Savela[1#], Alexander Winnett[1#], Anna E. Romano[1#], Michael K. Porter[1], Natasha Shelby[1], Reid Akana[1], Jenny Ji[1], Matthew M. Cooper[1], Noah W. Schlenker[1], Jessica A. Reyes[1], Alyssa M. Carter[1], Jacob T. Barlow[1], Colten Tognazzini[2], Matthew Feaster[2], Ying-Ying Goh[2], Rustem F. Ismagilov[1]*

3. California Institute of Technology, 1200 E. California Blvd., Pasadena, CA, USA 91125
4. City of Pasadena Public Health Department, 1845 N. Fair Oaks Ave., Pasadena, CA, USA 91103

*Correspondence to: rustem.admin@caltech.edu

[#] these authors contributed equally

medRxiv preprint doi: https://doi.org/10.1101/2021.04.02.21254771; this version posted April 7, 2021. The copyright holder for this preprint
(which was not certified by peer review) is the author/funder, who has granted medRxiv a license to display the preprint in perpetuity.
It is made available under a CC-BY 4.0 International license .

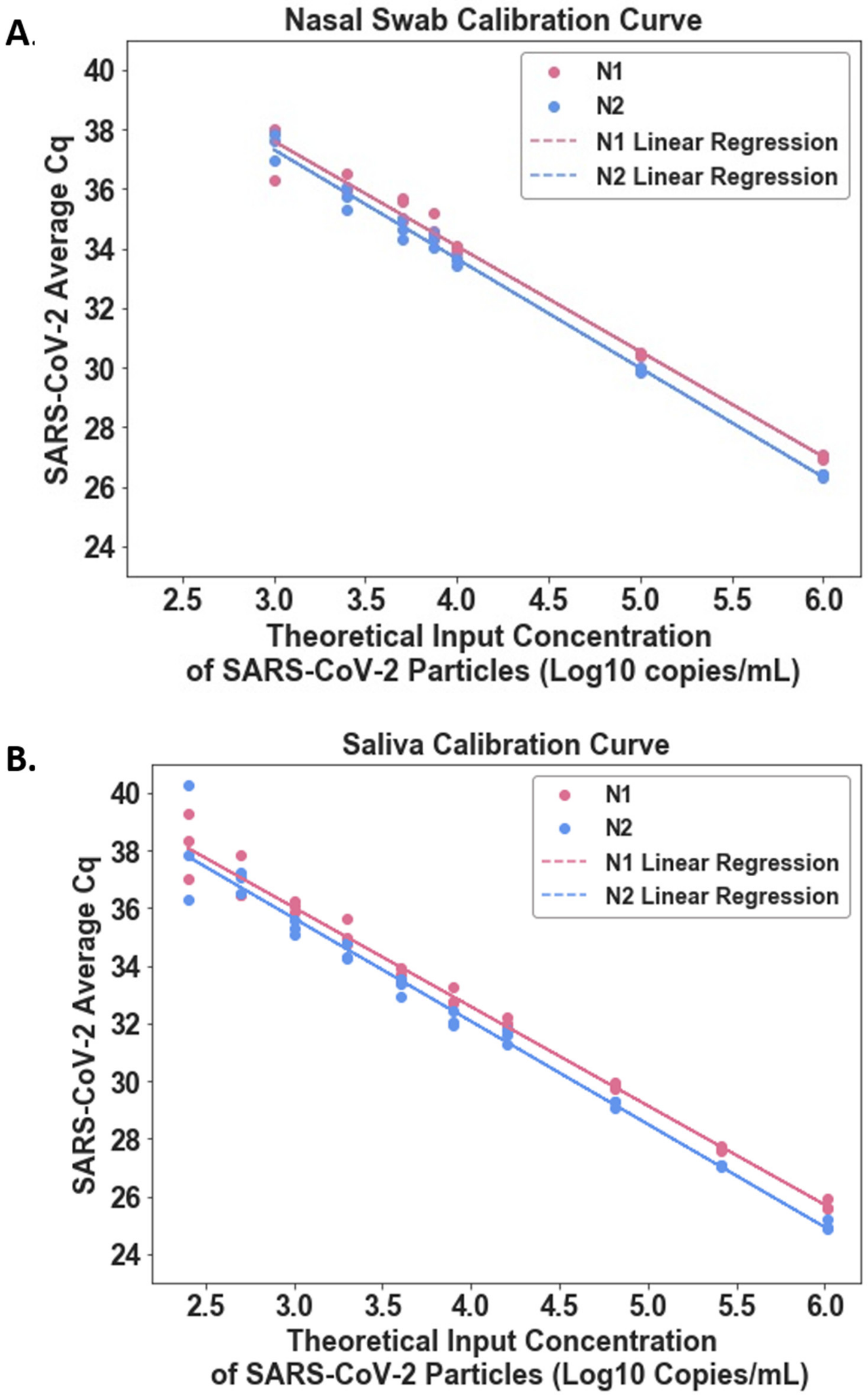

Figure S1. Calibration curve of SARS-CoV-2 inactivated particles to establish viral load conversion equations. Linear regression of RT-qPCR quantification cycle (Cq) for N1 (Red circle) and N2 (blue circles) genes at known concentrations of inactivated SARS-CoV-2 particles for saliva (A) or nasal swab (B) using this study's collection and laboratory workflows. Triplicate replicates per concentration were performed. Linear regression for N1 represented by red line and N2 represented by blue line. Linear regression R^2 values are 0.986 for N1 in nasal swabs, 0.994 for N2 in nasal swabs, 0.989 for N1 in saliva, and 0.979 for N2 in saliva.

medRxiv preprint doi: https://doi.org/10.1101/2021.04.02.21254771; this version posted April 7, 2021. The copyright holder for this preprint (which was not certified by peer review) is the author/funder, who has granted medRxiv a license to display the preprint in perpetuity.
It is made available under a CC-BY 4.0 International license .

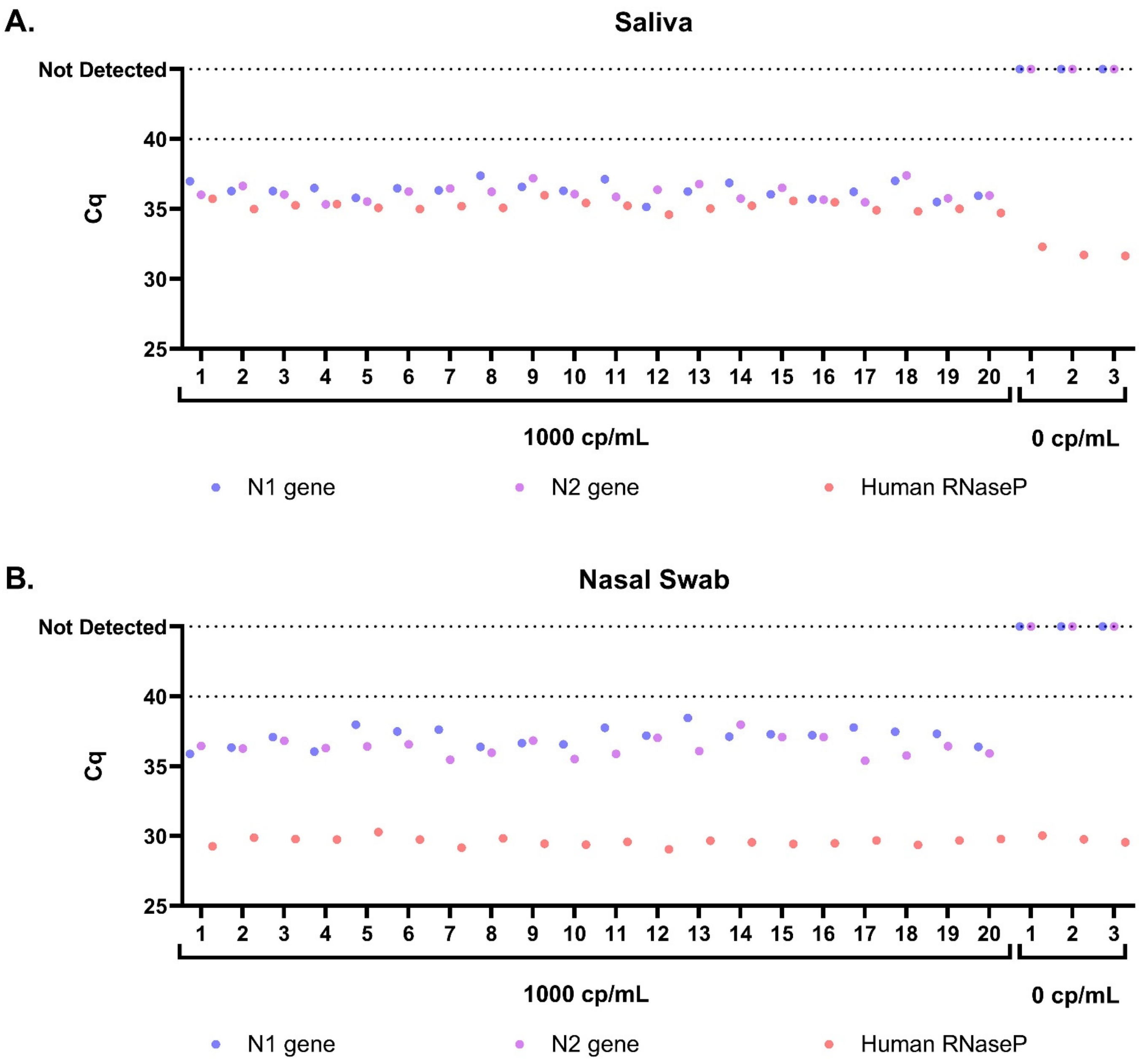

Figure S2. Limit of detection of saliva and nasal-swab RT-qPCR assays used in this study. RT-qPCR quantification cycle (Cq) for SARS-CoV-2 N1 gene (blue circle), N2 gene (purple circle), and human RNase P (orange circle) in 20 replicates of pooled matrix spiked with 1000 copies/mL (cp/mL) heat-inactivated SARS-CoV-2 RNA and 3 replicates of pooled matrix spiked with a buffer blank for saliva (A) and nasal swab (B) samples. Duplicate RT-qPCR reactions were performed for each extraction replicate and the averages are shown, with the following three exceptions: replicate 9 (saliva), in which the N1 gene only amplified in 1 of the duplicate runs (N2 in this run was positive, so per CDC EUA guidelines this run was interpreted as inconclusive), replicate 10 (nasal swab) in which the N2 gene only amplified in 1 of the duplicate runs (N1 in this run was positive, so this run was interpreted as inconclusive), and replicate 18 (nasal swab) in which the N1 gene only amplified in 1 of the duplicate runs (N2 in this run was positive, so this run was also interpreted as inconclusive). None of the samples spiked at 1000 copies/mL gave a negative detection result.

medRxiv preprint doi: https://doi.org/10.1101/2021.04.02.21254771; this version posted April 7, 2021. The copyright holder for this preprint (which was not certified by peer review) is the author/funder, who has granted medRxiv a license to display the preprint in perpetuity.
It is made available under a CC-BY 4.0 International license .

Table S1. Study participant demographic data. Figure 2 shows viral loads and symptoms data for the 4 participants for whom we observed transmission during their enrollment in the study.

	**Age Range (Years)**	**Sex**	**Race; Ethnicity**	**Reported Medical Conditions Associated with Increased Risk of Severe COVID-19[47]**
Fig. 2A	30-39	Male	Other; Mexican/Mexican American/Chicano (Salvadoran)	Diabetes
Fig. 2B	50-59	Male	Do not wish to respond; Mexican/Mexican American/Chicano	None
Fig. 2C	6-11	Female	White; Non-Hispanic	None
Fig. 2D	50-59	Male	American Indian or Alaskan Native, White; Other Hispanic, Latinx or Spanish origin	Obesity

medRxiv preprint doi: https://doi.org/10.1101/2021.04.02.21254771; this version posted April 7, 2021. The copyright holder for this preprint (which was not certified by peer review) is the author/funder, who has granted medRxiv a license to display the preprint in perpetuity.
It is made available under a CC-BY 4.0 International license .

Non-Corresponding Author Contributions

Listed alphabetically by last name

Reid Akana (RA): Assisted in literature analysis with ES, MKP, AW, MC; collaborated with AW in creating digital participant symptom surveys; assisted with data quality control/curation with JJ, NWS, NS; collaborated with ES, JJ to write data analysis/visualization code; created current laboratory information management system (LIMS) for specimen logging and tracking. Creation of iOS application for sample logging/tracking. Configured a SQL database for data storage. Created an Apache server and websites to view study data. Configured FTPS server to catalog PCR data. Wrote a Python package to access study data. Worked with ES, AW, AR to implement logic that prioritized specimen extraction order.

Jacob T. Barlow (JTB): Created initial specimen tracking database to aid in specimen logging and tracking. Maintenance of database and implementation of corrections. Feedback on manuscript draft.

Alyssa M. Carter (AMC): Received and logged specimens. Prepared reagents for and assisted with RNA extractions. Performed RT-qPCR and analyzed RT-qPCR data for both time series and screening experiments

Matthew M. Cooper (MMC): Collaborated with AW, MF, NS, YG, RFI, on study design and recruitment strategies. Co-wrote initial IRB protocol and informed consent with AW and NS; assisted in the writing of the enrollment questionnaire; developed laboratory sample processing workflow for saliva with AW and AER; performed sample processing on subset of samples; funding acquisition; collaborated with AR to write data processing/visualization code for observing household transmission events for active study participants. Contributor to the design of the calibration curve for saliva LOD experiments.

Matthew Feaster (MF): Co-investigator; collaborated with AW, MMC, NS, YG, RFI on study design and recruitment strategies; provided guidance and expertise on SARS-CoV-2 epidemiology and local trends.

Ying-Ying Goh (YYG): Co-investigator; collaborated with AW, MMC, NS, MF, RFI on study design and recruitment strategies; provided guidance and expertise on SARS-CoV-2 epidemiology and local trends.

Jenny Ji (JJ): Contributed to study design and study organization and implementation with NS and JAR; co-wrote enrollment questionnaire with NS and AW. Major contributor to curation of participant symptom data. Provided quality control of participant data with RA, NS, NWS. Major contributor to the symptom data analysis and visualization shown in Fig. 2.

Michael K. Porter (MKP): Performed specimen logging and QC, RNA extractions, RT-qPCR, data processing. Performed data acquisition and analysis for and made Figure S2 with AW. Prepared participant sample collection materials and helped with supplies acquisition. Assisted in literature analysis with ES, RA, AW.

Jessica A. Reyes (JAR): Study coordinator; collaborated with NS, AW, NWS, and RFI on recruitment strategies, translated study materials into Spanish, co-wrote informational sheets with AW and NS; created instructional videos for participants; enrolled and maintained study participants with NS and NWS.

Anna E. Romano (AER): Developed laboratory swab sample processing workflow with ES. Optimized extraction and ddPCR protocols working with vendor scientists. Created budgets and managed, planned, and purchased reagents and supplies ; developed and validated method for RT-qPCR and RT-digital droplet PCR analysis for saliva & swab samples with MMC, and AW. Performed specimen logging and QC, RNA extractions, RT-qPCR and RT-digital droplet PCR; Design of saliva calibration curve experiment. Analyzed ddPCR data for participant and calibration curve data included in Figure 1. Interpretation of sequence data with AW. Prepared Figure 1 and SI Figure S1 with ES and AW; Managing logistics for the expansion of the BSL-2+ lab space with ESS. Edited manuscript.

medRxiv preprint doi: https://doi.org/10.1101/2021.04.02.21254771; this version posted April 7, 2021. The copyright holder for this preprint
(which was not certified by peer review) is the author/funder, who has granted medRxiv a license to display the preprint in perpetuity.
It is made available under a CC-BY 4.0 International license .

Emily S. Savela (ESS): Coordinated the laboratory team and division of lab work, coordinated lab schedules to ensure completion of time-sensitive analyses of participant samples while complying with COVID-19 lab occupancy restrictions and biosafety requirements. Performed initial nasal swab workflow validation experiments with AER. Major contributor to workflow validation, methods, biosafety SOPs, and sample storage. Developed a plan for, and executed, the long term sample storage for efficient, safe, storage. Performed specimen logging and QC, RNA extractions, RT-qPCR, data processing, and conducted biosafety training. Performed the data curation and data analysis for Figure 2. Made Figure 2. Minor contributor to symptoms data analysis and visualization with JJ and RA for Figure 2. Experimental design and RNA extractions of the samples, to Figure 1A and minor contributor to Figure S1A with AER. Managing logistics for the expansion of the BSL-2+ lab space with AER and biohazardous waste pickups. Co-wrote the manuscript.

Noah W. Schlenker (NWS): Study coordinator; collaborated with NS, AW, JAR, and RFI on recruitment strategies; enrolled and maintained study participants with NS and JAR; study-data quality control, curation and archiving with RA, JJ, and NS.

Natasha Shelby (NS): Study administrator; collaborated with AW, MMC, RFI, YG, MF on initial study design and recruitment strategies; co-wrote IRB protocol and informed consent with AW and MMC; co-wrote enrollment questionnaire with AW and JJ; co-wrote participant informational sheets with AW and JAR; enrolled and maintained study participants with JAR and NWS; study-data quality control, curation and archiving with RA, JJ, and NWS; reagents and supplies acquisition; assembled Table S1; co-wrote and edited the manuscript.

Colten Tognazzini (CT): Coordinated the recruitment efforts at PPHD with case investigators and contact tracers; provided guidance and expertise on SARS-CoV-2 epidemiology and local trends.

Alexander Winnett (AW): Collaborated with MMC, NS, RFI, YG, MF on initial study design and recruitment strategies; cowrote IRB protocol and informed consent with MMC and NS; co-wrote enrollment questionnaire with NS and JJ; co-wrote participant informational sheets with NS and JAR and digital survey; developed and validated methods for saliva and nasal swab sample collection; developed and validated methods for RT-qPCR and RT-digital droplet PCR analysis for saliva and swab samples with AER, ESS, MMC; reagents and supplies acquisition; funding acquisition; developed laboratory sample processing workflow with AER, ESS, and MMC; performed specimen logging and QC, nucleic acid extraction, RT-qPCR, data processing – including experimental data generation for saliva calibration curve (Fig 1, Fig S1) designed with MMC and AER, establishment of nasal swab limit of detection (Fig S2), and viral load timeseries data (Fig 2) with ESS, AER, MKP, and AMC; interpreted sequencing data with AER; literature analysis with RA, ESS, and MKP; co-wrote sections of the manuscript outlined by ESS and RFI, edited the manuscript.
